# New assessment of *Anopheles* vector species identification using MALDI-TOF MS

**DOI:** 10.1186/s12936-020-03557-2

**Published:** 2021-01-09

**Authors:** Cécile Nabet, Abdoulaye K. Kone, Abdoulaye K. Dia, Moussa Sylla, Magali Gautier, Mohammed Yattara, Mahamadou A. Thera, Ousmane Faye, Leo Braack, Sylvie Manguin, Abdoul H. Beavogui, Ogobara Doumbo, Frédérick Gay, Renaud Piarroux

**Affiliations:** 1 Sorbonne Université, INSERM, Institut Pierre-Louis d’Epidémiologie et de Santé Publique, IPLESP, AP-HP, Groupe Hospitalier Pitié-Salpêtrière, Service de Parasitologie-Mycologie, 75013 Paris, France; 2 Malaria Research and Training Center (MRTC) UMI 3189 “Environnement, Santé, Sociétés”, CNRST, CNRS, UCAD, UGB, University of Sciences, Techniques and Technologies (USTTB), Bamako, Mali; 3 Laboratoire d’Ecologie Vectorielle et Parasitaire (LEVP), Université Cheikh Anta Diop, Dakar, Senegal; 4 Centre de Formation et de Recherche en Santé Rurale de Mafèrinyah, Conakry, Guinea; 5 APHM, Service de Parasitologie-Mycologie, Aix-Marseille Université, Marseille, France; 6 Malaria Consortium, Mahidol Faculty of Tropical Medicine, Bangkok, Thailand; 7 UP Institute for Sustainable Malaria Control, University of Pretoria, Pretoria, South Africa; 8 HydroSciences Montpellier (UMR-HSM), Institut de Recherche pour le Développement (IRD France), CNRS, 34093 Montpellier, France

**Keywords:** *Anopheles*, Malaria vectors, MALDI-TOF MS, Taxonomic identification, *Anopheles gambiae*, Head, Thorax, Legs

## Abstract

**Background:**

*Anopheles* species identification is essential for an effective malaria vector control programme. Matrix-assisted laser desorption ionization-time of flight (MALDI-TOF) mass spectrometry (MS) has been developed to identify adult *Anopheles* species, using the legs or the cephalothorax. The protein repertoire from arthropods can vary according to compartment, but there is no general consensus regarding the anatomic part to be used.

**Methods:**

To determine the body part of the *Anopheles* mosquitoes best suited for the identification of field specimens, a mass spectral library was generated with head, thorax with wings and legs of *Anopheles gambiae*, *Anopheles arabiensis* and *Anopheles funestus* obtained from reference centres. The MSL was evaluated using two independent panels of 52 and 40 *An. gambiae* field-collected in Mali and Guinea, respectively. Geographic variability was also tested using the panel from Mali and several databases containing added specimens from Mali and Senegal.

**Results:**

Using the head and a database without specimens from the same field collection, the proportion of interpretable and correct identifications was significantly higher than using the other body parts at a threshold value of 1.7 (p < 0.0001). The thorax of engorged specimens was negatively impacted by the blood meal after frozen storage. The addition of specimens from Mali into the database significantly improved the results of Mali panel (p < 0.0001), which became comparable between head and legs. With higher identification scores, the using of the head will allow to decrease the number of technical replicates of protein extract per specimen, which represents a significant improvement for routine use of MALDI-TOF MS.

**Conclusions:**

The using of the head of *Anopheles* may improve the performance of MALDI-TOF MS. Region-specific mass spectrum databases will have to be produced. Further research is needed to improve the standardization in order to share online spectral databases.

## Background

Approximately 70 mosquito species that belong to the genus *Anopheles* have the capacity to transmit parasites, such as *Plasmodium* species and *Wuchereria bancrofti,* agents of malaria and Bancroftian lymphatic filariasis, respectively. Thereby, *Anopheles* constitute a major public health concern [1, 2].

Traditional morphological identification with the use of dichotomous keys is the first step towards *Anopheles* vector species identification [3]. However, it requires technical skills and comprehensive training. It is also difficult for damaged specimens, new species, cryptic species, species with overlapping characteristics and cases of intraspecies morphological variation [4]. To overcome biased interpretations of species distributions and bionomics, molecular identification has been proposed as a complementary tool [5]. The most targeted gene for *Anopheles* species identification is the rDNA internal transcribed spacer region 2 (rDNA ITS2). However, specific primers are often required for species identification, such as that for the Sundaicus complex [6]. In addition, multiple gene sequences are often needed for unambiguous identification, especially due to poor availability of molecular reference databases [3, 7, 8].

Protein profiling using matrix-assisted laser desorption ionization time-of-flight mass spectrometry (MALDI-TOF MS) for arthropod identification is a promising tool [8, 9]. Several teams have built in-house databases to identify species of adults *Anopheles* by their MALDI-TOF spectra. Some of them used the legs to minimize the amount of material from specimen vouchers [10–14], whereas some other studies used the cephalothorax [15, 16]. Consequently, there is no general consensus regarding the optimal anatomic part to be used. The protein repertoire from arthropods has been shown to vary according to compartment [8, 17]. There is a need to establish a standardized and optimized protocol determining which body part produces the most reproducible and specific mass spectra protein profile [8, 9]. In addition, it is important to evaluate the influence of geographic variability on identification results, as it may lead to protein variability [10, 16].

The aim of this study was to determine the anatomic part of *Anopheles* adult mosquitoes, both males and females, best suited for the identification of field specimens. A mass spectral library (MSL) was generated using different mosquito body parts, for both males and females, obtained from reference centres. The MSL was evaluated using two independent panels of field-collected specimens from Mali and Guinea. Geographic variability was tested using several databases containing additional specimens from Mali and Senegal.

## Methods

### Study design

**Fig. 1:**
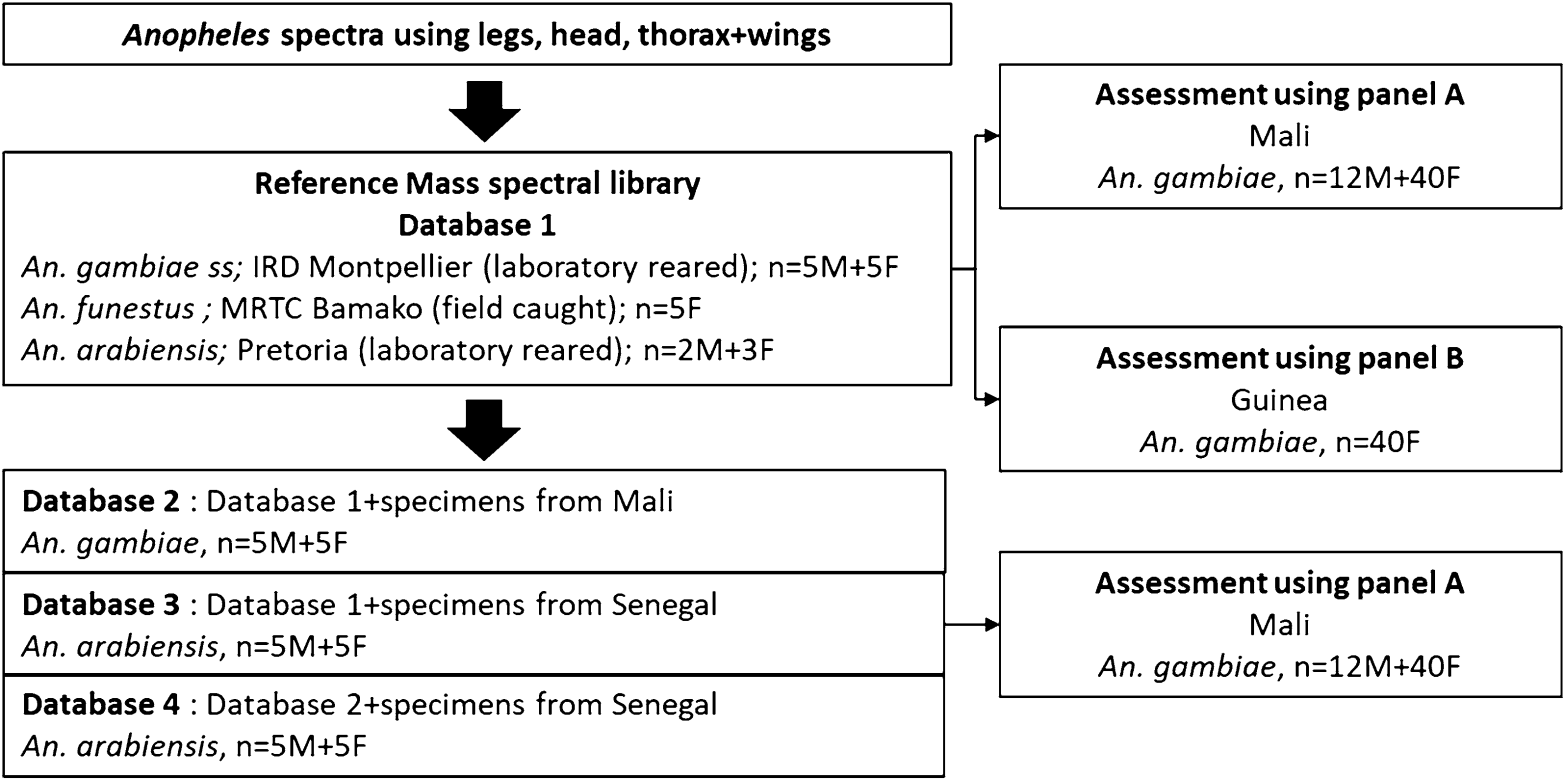
Study flowchart of MALDI-TOF MS assessment for the identification of *Anopheles* species. *M* male, *F* female

A reference MSL (database 1) was created using non-engorged laboratory-reared and field-collected *Anopheles* obtained from collections of reference centres (Table 1; Fig. 1). To evaluate the impact of body part selection on the accuracy of species identification, a panel of 52 field-collected *Anopheles gambiae* including 12 engorged females from Mali (panel A) was tested against database 1. To further evaluate the reproducibility of the results, an extra panel of 40 field-collected *An. gambiae* including 6 engorged females from Guinea (panel B) was also tested against database 1. To evaluate the impact of the database species composition and geographic origin, 3 extra databases were created. A second database (database 2) was created by adding 10 field-collected *An. gambiae* from Mali to database 1 that were not previously included in panel A. Database 3 and database 4 were created by adding 10 field-collected *Anopheles arabiensis* from Senegal to databases 1 and 2, respectively. These extra databases were tested using panel A. Field specimens from Mali were adult mosquitoes collected indoors between July and August 2016 using human landing catches and aspiration of resting fauna after insecticide spraying in the villages of Doneguebougou (Kati district), Bancoumana (Kati district), Bougoula-Hameau (Sikasso district) and Sotuba (Bamako district). In Senegal, larvae were collected in a field in Wakhinane-Nimzatt (Guediawaye district) in November 2018 and were reared to the adult stage. In Guinea, adult mosquitoes were collected outdoors in August 2019 using human landing catches in the village of Senguelen (Maferinyah district). Before analysis, specimens were stored dry frozen at − 20 °C after a shipping delay at ambient temperature that did not exceed 3 weeks. All specimens were sorted using morphological identification keys [18] and identified to the species level by PCR sequencing of the rDNA ITS2 [4, 19]. *Anopheles arabiensis* and *An. gambiae*, are cryptic species belonging to the Gambiae complex and were distinguished using the ITS2 marker. However, the two taxonomic species *An. gambiae* and *Anopheles coluzzii* were not distinguishable using the ITS2 marker. Only the intergenic spacer (IGS) marker is able to differentiate these two taxonomic species, although it was not analysed in this study [20]. The two close species were, therefore, designated as *An. gambiae*. The storage conditions and mean delay until analyses vary between samples and are represented in Table 1. The different storing conditions, environmental conditions and geographical origins will be useful to show which anatomic part is less prone to degradation and exhibit the most robust mass spectra.

**Table 1:** Characteristics of *Anopheles* used to create databases and panels

Species	Country	Source	Collection year, storage	Mean delay until analyses	No. males	No. females	No. engorged females	Anatomic part to be tested	Database number
*Anopheles gambiae*	Kenya, Kisumu	Lab reared, IRD^a^ Montpellier	2015, − 80 °C	1 year	5	5	0	Head, thorax, legs	1, 2, 3, 4
*Anopheles funestus*	Mali	Field caught	2016, N_2_, then − 20 °C	2 months	0	5	0	Head, thorax, legs	1, 2, 3, 4
*Anopheles arabiensis*	South Africa	Lab reared, University of Pretoria	2018, silica gel, ambient T, then − 20 °C	3 weeks	2	3	0	Head, thorax, legs	1, 2, 3, 4
*Anopheles gambiae*	Mali	Field caught	2016, N_2_, then − 20 °C	2 years	5	5	1	Head, thorax, legs	2, 4
*Anopheles arabiensis*	Senegal	Field caught	2018, silica gel, ambient T, then − 20 °C	3 weeks	5	5	0	Head, thorax, legs	3, 4
*Anopheles gambiae*	Mali	Field caught	2016, N_2_, then − 20 °C	2 months	12	40	12	Head, thorax, legs	Panel A
*Anopheles gambiae*	Guinea	Field caught	2019, silica gel, ambient T, then − 20 °C	2 months	0	40	6	Head, thorax, legs	Panel B

^a^IRD: French National Research Institute for Sustainable Development

### Molecular identification of *Anopheles*

After dissection, abdomens were incubated for 24 h at room temperature in 800 µL of EasyMAG lysis buffer (BioMérieux, Marcy l’Étoile, France). Samples were homogenized into a MagNALyser Instrument (Roche Diagnostics, Meylan, France) using ceramic bead tubes. Nucleic acid extraction was performed using a NucliSENS EasyMAG system (BioMérieux, Marcy l’Étoile, France) with an elution volume of 50 µL. The ITS2 region was amplified using the ITS2A and ITS2B primers [4]. The primer sequences were as follows: ITS2A 5′-TGTGAACTGCAGGACACAT-3′ and ITS2B 5′-TATGCTTAAATTCAGGGGGT-3′. The 32 µL PCR mixture contained 13.75 μL of 1X Light Cycler Mix (Roche Diagnostics, Meylan, France), 1 μL of 10 µmol/L each forward and reverse primers, and 2 μL of DNA template. The thermocycling conditions were as follows: 94 °C for 5 min; 30 cycles of denaturation at 94 °C for 1 min, annealing at 53 °C for 1 min, and extension at 72 °C for 2 min; and a final extension at 72 °C for 5 min. The PCR products were sequenced directly (with one of the PCR primers) using Sanger sequencing on an ABI 3730xl DNA Analyzer platform (PE Applied Biosystems, Warrington, UK). Sequence chromatograms were visually inspected, and consensus sequences were generated using Seaview v4 software. Multiple sequence alignment was performed using the Clustal W and G-blocks tools implemented in Seaview v4. Maximum likelihood analysis with PhyML (1000 iterations for bootstrapping, GTR model) [21] was processed by implementing the sequences of the field specimens to the sequences of *Anopheles* specimens morphologically identified in reference centres from the MSL (GenBank accession numbers MN335037, MN335038, and MN335049 to MN335053). Field specimens’ sequences of *An. gambiae* (GenBank accession numbers MN334973 to MN335036 for Mali specimens and MN830441 to MN830480 for Guinea specimens) and *An. arabiensis* (GenBank accession numbers MN335039 to MN335048) were assigned at the species level when they clustered within the clade of the species reference sequences (Additional file 1: Fig. S1).

### Sample preparation for MALDI-TOF MS analysis

After dissection, heads, legs, and thoraces with wings were processed separately. They were put into individual 1.5-mL microcentrifuge tubes and rinsed in 70% ethanol for 10 min. Tubes were centrifuged at 13,000 rpm for 10 min, and the supernatant was discarded. After a second centrifugation (13,000 rpm, 2 min), the remaining ethanol solution was then eliminated using a micropipette and left to evaporate. Protein extraction was performed after the addition of 10 µL of 70% formic acid. After manual homogenization with a micropipette, the homogenate was incubated for 5 min. Then, 10 µL of 100% acetonitrile was added and incubated for 5 min. The homogenate was centrifuged (13,000 rpm, 2 min), and 1 µL of the supernatant of each sample containing the protein extract was deposited onto a steel target plate (Bruker Daltonics, Wissembourg, France). Once dried, the deposits were covered with a 1-µL alpha-cyano-4-hydroxycinnamic acid (HCCA) matrix prepared in 50% acetonitrile and 2.5% trifluoroacetic acid and 47.5% of HPLC grade water (final concentration of 10 mg/mL). To ensure the reproducibility of the results, a total of ten replicates were spotted for each specimen to be included in the database, and a total of four replicates were spotted for each specimen of the panel to be tested, as previously published [22, 23].

### Mass spectrum acquisition

Mass spectra were acquired with a Microflex LT (Bruker France SAS) using the default acquisition parameters. The spectra were acquired in linear mode in the ion-positive mode at a laser frequency of 60 Hz and mass range of 2–20 kDa. Each spectrum was obtained from 240 laser shots in 6 regions of each spot. The data were automatically acquired using AutoXecute in FlexControl v3.4 software (Bruker France SAS) and exported into MALDI Biotyper v4.1 software (Bruker France SAS) for data processing with the default parameters and spectrum analysis.

### Mass spectral library construction

To construct an MSL, one reference spectrum was created for each specimen and for each anatomic part. Each reference spectrum was an average spectrum also called a main spectrum profile (MSP) resulting from 10 raw spectra, obtained from a spotting of ten replicates of protein extract. In database 1, a total of 20 specimens led to 600 spectra and 60 MSPs. Five to ten specimens by species were included in databases, allowing to assess the intra-species variability. The compactness of database 1 for MALDI Biotyper v4.1 identification of *Anopheles* species was evaluated by computing a crosswise comparison in which the 600 spectra of each specimen were compared with the 60 MSPs of all *Anopheles* included in database 1 [24]. When a list of unknown spectra is compared with the MSPs of a reference database using MALDI Biotyper v4.1 software, a score value ranging from 0 to 3 logarithmic units is automatically generated along with a list of species matches. The higher the log(score) value (LSV) is, the higher the probability that the unknown spectrum belongs to the same group as the corresponding reference MSP. During crosswise comparison, the first hits corresponding to cross-identification with any spectrum of the same specimen were eliminated, and the second or third hits of cross-identification were selected for the analysis. The LSV threshold for an interpretable identification result was defined at 1.7 because it is a commonly used threshold for arthropod vector species identification using MALDI-TOF [13, 22]. The impact of various LSV thresholds from 1.7 to 2 was assessed, and modifying the threshold did not impact the main results presented in the study (Additional file 2: Fig. S2).

### Mass spectral library versus panel

Each anatomic part, including heads, legs and thoraces with wings, was processed for MALDI-TOF MS identification, following the same protocol as that applied for specimens of the MSL. Each of the four raw spectra of the panels obtained from each anatomic part was analysed against databases 1 (panels A and B), 2, 3 and 4 (panel A). As previously published [22, 23], only the replicate with the highest LSV was selected, and the identification corresponded to the first hit obtained for this replicate. MALDI-TOF MS identifications were compared to molecular identifications for every specimen. For the legs and head, distributions of identification log(scores) were compared, according to number of deposits of protein extract per specimen, using Panel A versus Database 2. The best log (score) was recorded according to the using of one, two, three or four spots of protein extract of legs and head, applying different values of log(score) threshold from 1.7 to 2. The results of combinations of spots were analysed chronologically from the first to the fourth sample of protein extract deposited onto the target plate.

### Mass spectral analysis

To assess spectral variation within the set of spectra of database 1, panel A and panel B, a composite correlation index (CCI) that considers peak positions, peak intensity distribution and peak frequency was computed using MALDI Biotyper v4.1 software with default settings (mass range, 3.0–12.0 kDa; resolution 4; eight intervals; autocorrection off). The matrix of the correlation indexes was represented as a heat map grid (index variation from 0 to 1). The levels of mass spectrum correlations are indicated from red to blue, revealing relatedness and incongruence between spectra, respectively. To assess the mass spectrum relationship to one another, an unsupervised clustering analysis (dendrogram) was performed according to mass protein profiles (m/z, intensity) using MALDI Biotyper v4.1 software. The calculation mode was set to the default settings, the distance was measured by correlation, the linkage by the mean and the score threshold value for a single organism was 300 arbitrary units and 0 arbitrary units for related organisms. The closeness of one *Anopheles* spectrum to other spectra was reflected by an arbitrary distance level.

### Statistical analysis

Since most of the quantitative variables differ significantly from a normal distribution (histograms, Q-Q plots, normality tests), non-parametric exact tests were conducted. To compare a quantitative variable (i.e., distributions of LSVs) between 3 paired samples (i.e., head versus thorax versus legs), the Friedman test (with 10,000 Monte Carlo simulations) was used followed by pairwise comparisons taking into account the alpha risk inflation. All tests were interpreted in a 2-sided way. The Mann & Whitney test was applied to compare a quantitative variable (i.e., distributions of LSVs) between two independent samples (body parts of database 1 versus database 2). Fisher’s exact test was used to compare a binomial variable (i.e., proportions of correct species, LSV ≥ 1.7) between two independent samples (i.e., body parts, databases, panels).

## Results

### Impact of body part

#### Mass spectrum protein profiles

**Fig. 2:**
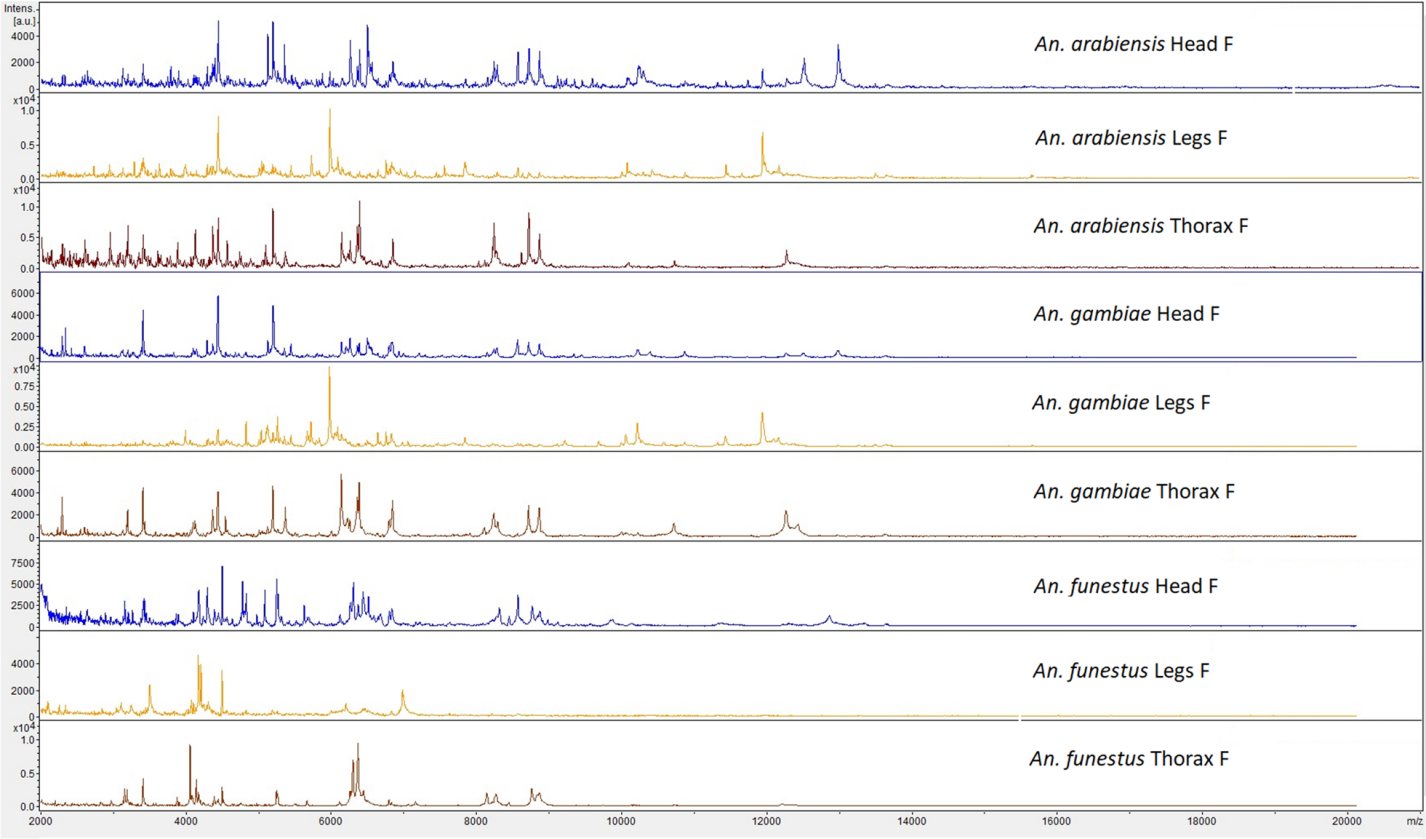
Representative mass spectrum protein profiles of the different body part of each *Anopheles* species included in the mass spectra library. *A.u* arbitrary units, *m/z* mass to charge ratio in Daltons, *F* female

The mass spectrum protein profiles of head, thorax and legs of each *Anopheles* species included in the MSL are shown in Fig. 2. The protein profiles differed importantly between each body part, for each species. The spectra of legs displayed less peaks of high intensity than the spectra of head and thorax. Between *An. arabiensis* from South Africa and *An. gambiae* from Kenya, species of the Gambiae complex, shared peaks were observed for all the anatomic parts.

#### Reproducibility of mass spectra

**Fig. 3:**
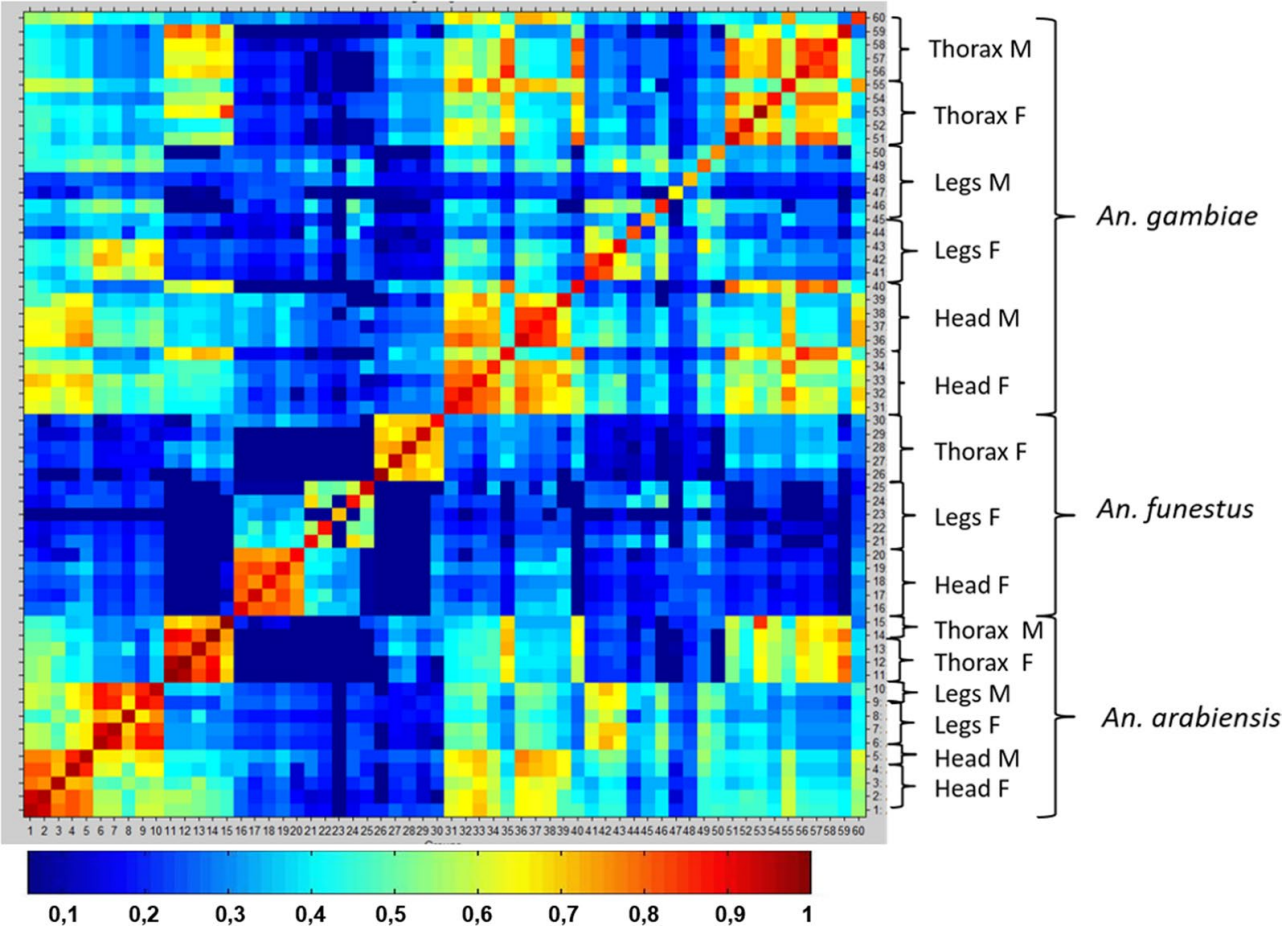
Composite correlation index (CCI) heat map grid of mass spectrum protein profiles, database 1, *n* = 60. Levels of mass spectral reproducibility are indicated in blue and red, revealing incongruence and relatedness between spectra, with a correlation index variation between 0 and 1, respectively. The coloured squares of the central diagonal reflect the degree of reproducibility of each mass spectrum when compared to itself. Around the central diagonal, spectra from various specimens of the same species were compared as well as spectra from different species. The CCI matrix was calculated using MALDI Biotyper v4.1 software with default settings

Mass spectra reproducibility differed between anatomic parts (Fig. 3; Additional file 3: Fig. S3). Database 1 mass spectra displayed a high reproducibility level (Fig. 3). The highest level of correlation was observed between spectra from the same species and the same body part. Within the same species, the highest correlations between spectra were observed for the heads and thoraces, and the lowest correlations were observed for the legs. Only *An. arabiensis* exhibited highly reproducible spectra for every body parts. The spectra of *An. arabiensis* were acquired after three weeks of storage at − 20 °C, whereas the spectra of *Anopheles funestus* were acquired after two months at − 20 °C and that of *An. gambiae,* after one year at − 80 °C, suggesting an impact of storage conditions. The high intraspecies specificity of the mass spectra was confirmed by the low correlations between the spectra of *An. funestus* and *An. gambiae* or *An. arabiensis*. As expected, between-spectra cross-correlations were observed for the cryptic species of the Gambiae complex, *An. gambiae* and *An. arabiensis*. Compared to database 1, the reproducibility levels of mass spectra from field-collected *An. gambiae* (panel A and panel B) were highly heterogeneous and lower (Additional file 3: Fig. S3). The head spectra from field-collected *An. gambiae* were the most reproducible compared to the thorax and the legs.

#### Distributions of identification log(scores)

**Fig. 4:**
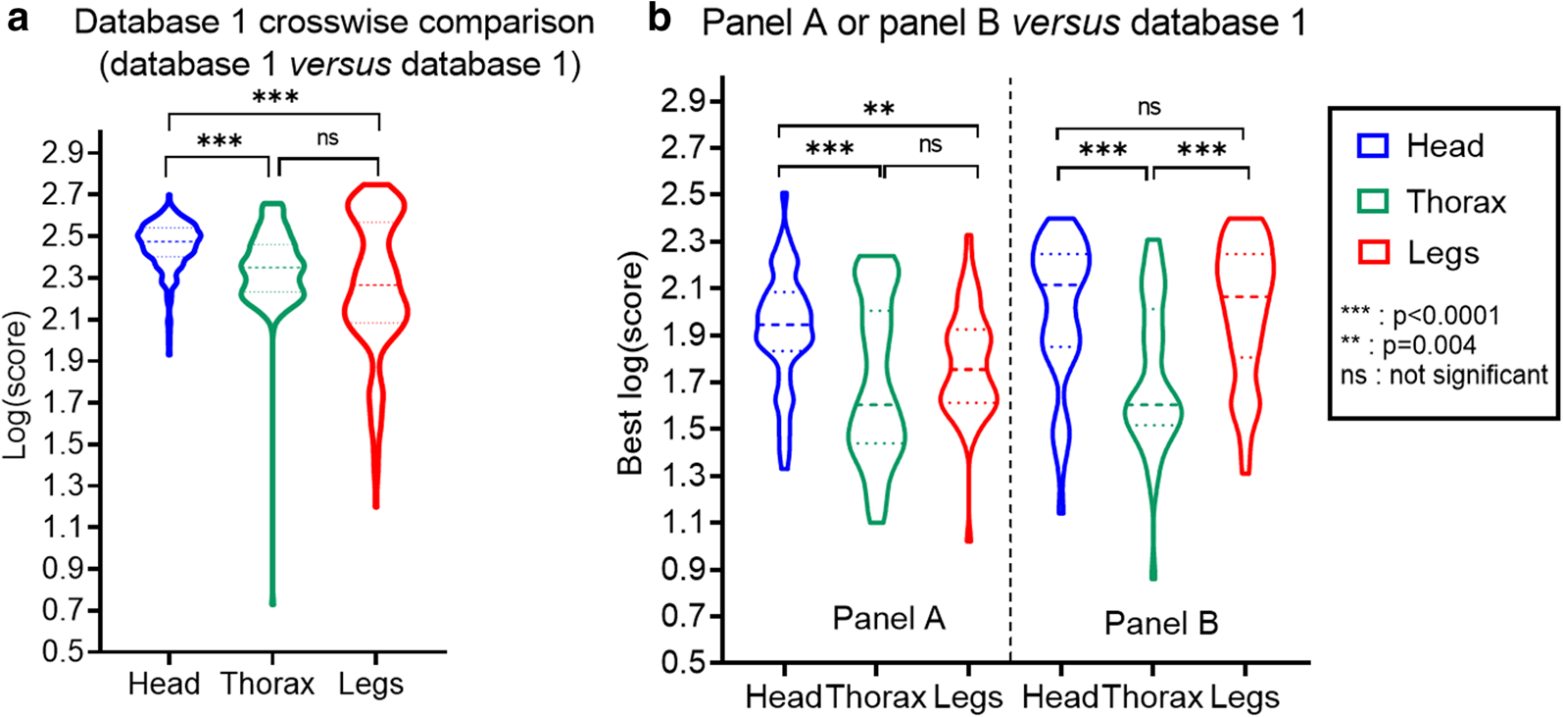
Distribution of spectrum log(scores) from heads, thoraces and legs. Crosswise comparison of database 1 (database 1 versus database 1, n = 200 spectra) after exclusion of the LSV results corresponding to spectra that belong to the same specimen (**a**). Panel A or panel B versus database 1, n = 52 best log(score) and n = 40 best log(score), respectively (**b**). Violin plots showing the distribution taking into account the densities of the points for the different log(score) values. The median score is represented with dashes, and the quartiles are represented by dashed lines

During database 1 crosswise comparison (Fig. 4a), spectra from heads, thoraces and legs exhibited high median LSV (LSV = 2.47, LSV = 2.35, and LSV = 2.26, respectively), indicating a high quality of mass spectra even if legs and thorax showed outlier spectra. In contrast, testing panel A versus database 1 (Fig. 4b), spectra from heads, thoraces and legs exhibited lower median LSV (LSV = 1.94, LSV = 1.60, and LSV = 1.75, respectively). Testing panel B versus database 1 (Fig. 4b), spectra from heads and legs exhibited higher median LSV compared to panel A (LSV = 2.11 and LSV = 2.06, respectively), but thorax median LSV was similar (LSV = 1.60).

The distribution of LSVs of head spectra differed significantly from that of LSVs of the thorax spectra (p < 0.0001), during database 1 crosswise comparison (Fig. 4a) and testing panel A and panel B versus database 1 (Fig. 4b). This was also observed when comparing the head to the legs, during database 1 crosswise comparison (p < 0.0001) and testing panel A versus database 1 (p = 0.004). However, testing panel B versus database 1, no significant difference in LSV distribution was observed between the head and legs (p = 0.4).

#### Identification results using database 1

During the query of two independent panels (panels A + B) versus database 1, the proportion of interpretable (LSV ≥ 1.7) and correct identifications was significantly higher using the head than using the thorax or the legs (p < 0.0001 or p < 0.0001, respectively) (Table 2; Additional file 4: Fig. S4). Using the head, 64.13% of specimens (59/92) exhibited correct identification versus 27.17% (25/92) using the thorax and 29.35% (27/92) using the legs. The proportion of specimens with an LSV ≥ 1.7 was higher using the head, accounting for 83.7% (77/92) of specimens versus 42.39% (39/92) for the thorax and 67.39% (62/92) for the legs (p < 0.0001 and p = 0.01, respectively).

**Table 2:** Identification results, panel A (n = 52), panel B (n = 40) and panels A + B (n = 92) versus database 1

	Head	Thorax	Legs
Panel A			
Correct species^a^, no. specimens (%)	32/52 (61.54)	11/52 (21.15)	16/52 (30.77)
Wrong species^b^, no. specimens (%)	11/52 (21.15)	12/52 (23.08)	14/52 (26.92)
Absence of identification, LSV < 1.7, no. specimens (%)	9/52 (17.31)	29/52 (55.77)	22/52 (42.31)
Panel B			
Correct species^a^, no. specimens (%)	27/40 (67.5)	14/40 (35)	11/40 (27.5)
Wrong species^b^, no. specimens (%)	7/40 (17.5)	2/40 (5)	21/40 (52.5)
Absence of identification, LSV < 1.7, no. specimens (%)	6/40 (15)	24/40 (60)	8/40 (20)
Panels A + B			
Correct species^a^, no. specimens (%)	59/92 (64.13)	25/92 (27.17)	27/92 (29.35)
Wrong species^b^, no. specimens (%)	18/92 (19.57)	14/92 (15.22)	35/92 (38.04)
Absence of identification, LSV < 1.7, no. specimens (%)	15/92 (16.30)	53/92 (57.61)	30/92 (32.61)

^a^Proportion of interpretable (LSV ≥ 1.7) and correct identifications among all the tested specimens

^b^Proportion of interpretable (LSV ≥ 1.7) and wrong identifications among all the tested specimens

Testing panel A versus database 1, the proportion of correct identifications accounted for 61.54% (32/52) of specimens using the head versus only 21.15% (11/52) using the thorax and 30.77% (16/52) using the legs (p < 0.0001 and p = 0.003, respectively). The proportion of specimens with an LSV ≥ 1.7 was higher using the head, accounting for 82.69% (43/52) of specimens versus 44.23% (23/52) for the thorax and 57.69% (30/52) for the legs (p < 0.0001 and p = 0.009, respectively).

Testing panel B versus database 1, the proportion of correct identifications accounted for 67.50% (27/40) of specimens using the head versus only 35% (14/40) using the thorax and 27.5% (11/40) using the legs (p = 0.007 and p = 0.0007, respectively). The proportion of specimens with an LSV ≥ 1.7 was higher using the head than using the thorax, accounting for 85% (34/40) of specimens versus 40% (16/40) for the thorax (p < 0.0001). However, no significant difference was observed between the head and the legs and 80% (32/40) of specimens had an LSV ≥ 1.7 using the legs (p = 0.8).

### Impact of blood meal

**Fig. 5:**
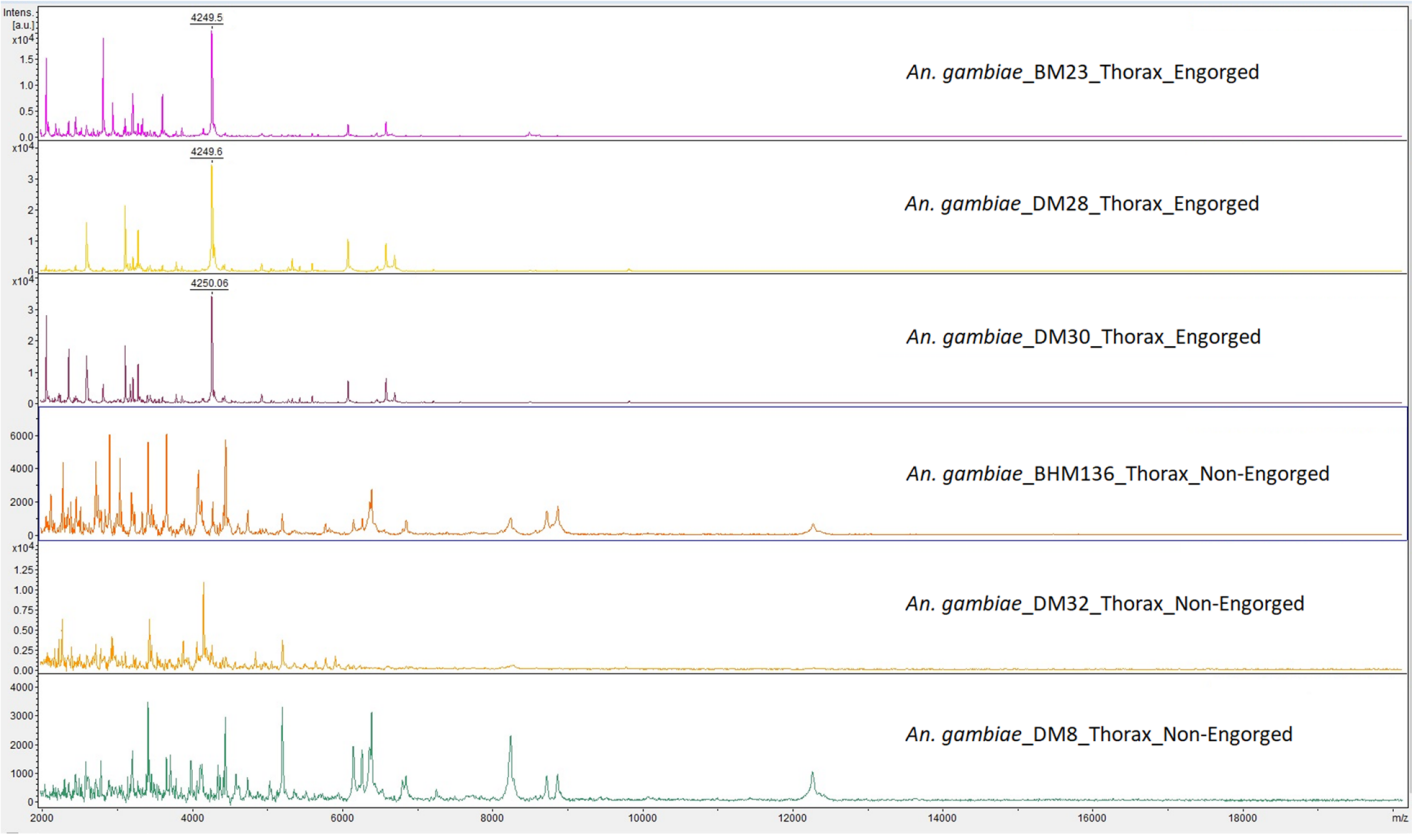
Representative mass spectrum protein profiles of thorax from engorged and non-engorged *Anopheles gambiae* specimens of panel A from Mali. *A.u* arbitrary units, *m/z* mass to charge ratio in Daltons, *F* female

An impact of the blood meal on thorax mass spectra was suspected as 72.5% (29/40) of the thorax spectra from females had an LSV < 1.7, but no thorax spectra from males (0/12) using panel A versus database 1. Among the thorax spectra from females, the totality of the engorged *Anopheles* (12/12) had an LSV < 1.7, in contrast to 60.71% (17/28) of non-engorged female specimens (p = 0.02). This significant variation in the proportion of specimens with an LSV < 1.7 between engorged and non-engorged females was observed only for the thoraces but not for the legs and head. Indeed, using the legs, between engorged and non-engorged females, the proportions of specimens with an LSV < 1.7 were, respectively, of 66.67% (8/12) and 46.43% (13/28), (p = 0.3). Using the head, between engorged and non-engorged females, this accounted for 16.67% (2/12) and 25% (7/28), (p = 0.7), respectively. Thorax spectra protein profiles of *An. gambiae* specimens from Mali differed importantly between engorged and non-engorged, confirming the impact of the blood meal (Fig. 5). Spectra from engorged specimens exhibited a specific pattern with a peak of high intensity (*m*/*z* 4250), absent of non-engorged specimens. As only non-engorged specimens were included into the database 1, this can explain the absences of identification for engorged specimens. Indeed, the totality of engorged specimens of the panel A could not be identified using the thorax and accounted for 41.38% (12/29) of the absences of identification due to an LSV < 1.7, whereas they represented only 23.07% (12/52) of the tested specimens. Using the legs or the head, engorged specimens respectively accounted for 36.36% (8/22) and 22.22% (2/9) of the absences of identification.

### Cross-matching between body parts and sex

Using database 1, panel A spectra preferentially matched spectra of the same body part (Additional file 5: Fig. S5). Of the tested spectra of head, thorax and legs from panel A, a total of 96 spectra had an LSV ≥ 1.7. Of the 96 spectra with an LSV ≥ 1.7, 81 (84.4%) matched the spectra resulting from the same body part. Overall cross-matching between body parts was observed for only 15.6% (15/96) of spectra. However, when considering legs spectra, cross-matching with head or thorax spectra accounted for 46.7% (14/30) of the spectra**.** A preferential matching with the same sex was observed for 63.5% (61/96) of spectra.

With database 1, the using of multiple anatomic parts in the panel A, by combining the identification results of “head + legs”, “head + thorax” or “head + legs + thorax”, did not increase the proportion of correct identifications compared to the using of the head alone (p = 1, p = 0.5, and p = 0.7, respectively) (Additional file 6: Fig. S6). Similarly, the proportion of specimens with an LSV ≥ 1.7 did not increase (p = 0.8, p = 1, and p = 0.8, respectively). However, compared to the using of the association “thorax + legs”, the proportion of correct identifications and the proportion of specimens with an LSV ≥ 1.7 were significantly higher using the head alone (p = 0.006 and p = 0.03, respectively).

### Impact of database species composition and geographic origin

#### Distributions of identification log(scores) using panel A

The using of database 2 that included Mali specimens against panel A from Mali led to a significant increase in the LSVs compared to the using of database 1 (p < 0.0001) (Additional file 7: Fig. S7). The distribution of LSVs of head spectra also differed significantly from that of the thorax spectra and that of legs spectra (p = 0.02 and 0.005, respectively). However, no significant difference in LSVs distribution was observed between the thorax and legs (p = 0.9).

#### Identification results using databases 1, 2, 3 and 4 and panel A

The number of specimens having correct species identification, error of species identification and absence of identification due to an LSV < 1.7 differed significantly according to the database for all anatomic parts (p < 0.0001) (Table 3, Additional file 8: Fig. S8). From database 1 to database 2, the inclusion of *An. gambiae* specimens from Mali significantly increased the proportion of correct identifications from 61.54% (32/52) to 98.08% (51/52) using the head, from 21.15% (11/52) to 80.77% (42/52) using the thorax and from 30.77% (16/52) to 96.15% (50/52) using the legs (p < 0.0001). The proportions of mismatch between *An. gambiae* and *An. arabiensis* increased but not significantly after the addition of *An. arabiensis* specimens from Senegal, from database 1 to database 3 using the legs and head and from database 2 to database 4 using the head (Table 3).

**Table 3:** Identification results, panel A versus database 1, database 2, database 3 or database 4, n = 52

	Database 1	Database 2	Database 3	Database 4
Head				
Correct species^a^, no. specimens (%)	32/52 (61.54)	51/52 (98.08)	28/52 (53.85)	48/52 (92.31)
* An. arabiensis* instead of *An. gambiae*^b^, no. specimens (%)	10/52 (19.23)	1/52 (1.92)	15/52 (28.85)	4/52 (7.69)
* An. funestus* instead of *An. gambiae*^b^, no. specimens (%)	1/52 (1.92)	0/52	1/52 (1.92)	0/52
Absence of identification, LSV < 1.7, no. specimens (%)	9/52 (17.31)	0/52	8/52 (15.38)	0/52
Thorax				
Correct species^a^, no. specimens (%)	11/52 (21.15)	42/52 (80.77)	11/52 (21.15)	42/52 (80.77)
* An. arabiensis* instead of *An. gambiae*^b^, no. specimens (%)	12/52 (23.08)	6/52 (11.54)	12/52 (23.08)	6/52 (11.54)
* An. funestus* instead of *An. gambiae*^b^, no. specimens (%)	0/52	0/52	0/52	0/52
Absence of identification, LSV < 1.7, no. specimens (%)	29/52 (55.77)	4/52 (7.69)	29/52 (55.77)	04/52 (7.69)
Legs				
Correct species^a^, no. specimens (%)	16/52 (30.77)	50/52 (96.15)	12/52 (23.08)	50/52 (96.15)
*An. arabiensis* instead of *An. gambiae*^b^, no. specimens (%)	13/52 (25)	0/52	19/52 (36.54)	0/52
* An. funestus* instead of *An. gambiae*^b^, no. specimens (%)	1/52 (1.92)	0/52	1/52 (1.92)	0/52
Absence of identification, LSV < 1.7, no. specimens (%)	22/52 (42.31)	2/52 (3.85)	20/52 (38.46)	2/52 (3.85)

^a^Proportion of interpretable (LSV ≥ 1.7) and correct identifications among all the tested specimens

^b^Proportion of interpretable (LSV ≥ 1.7) and wrong identifications among all the tested specimens

**Fig. 6:**
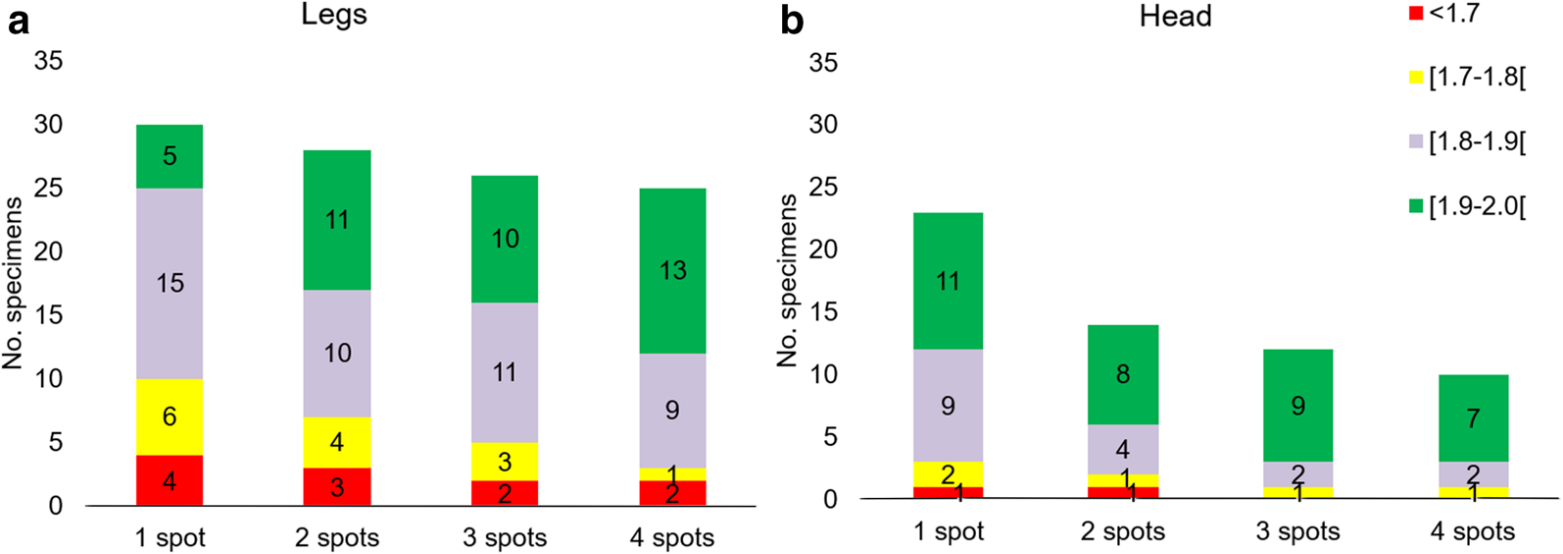
Distribution of identification log(scores) according to number of spots per specimen using panel A versus database 2. The best log (score) was recorded according to the using of one, two, three or four spots of protein extract of legs (**a**) and head (**b**). The results of combinations of spots were analysed chronologically from the first to the fourth sample of protein extract deposited onto the target plate

With database 2 and using four replicates of protein extract and an LSV threshold of 1.7, proportions of correct identification were comparable between the head (98.08%) and legs (96.15%) (p = 1). With database 4, the difference was higher between the head (92.31%) and legs (96.15%) but was also not significant (p = 0.7). However, the distributions of identification log(scores) differed significantly between the two body parts when using less than four replicates of protein extract per specimen and various LSV thresholds from 1.7 to 2 (Fig. 6). Using only one spot, the head distribution of identification log(scores) was equivalent to that of the legs but using four spots. Indeed, with the head, the number of absences of identification was almost equivalent using four spots compared to using one spot, at an LSV threshold of 1.7 (n = 0 and n = 1, respectively) and 1.8 (n = 1 and n = 3, respectively). In contrast, with the legs, the number of absences of identification increased, at an LSV threshold of 1.7 (n = 2 and n = 4, respectively) and 1.8 (n = 3 and n = 10, respectively).

### Comparison of spectrum protein profiles of *An. gambiae* from Kenya, Mali and Guinea

**Fig. 7:**
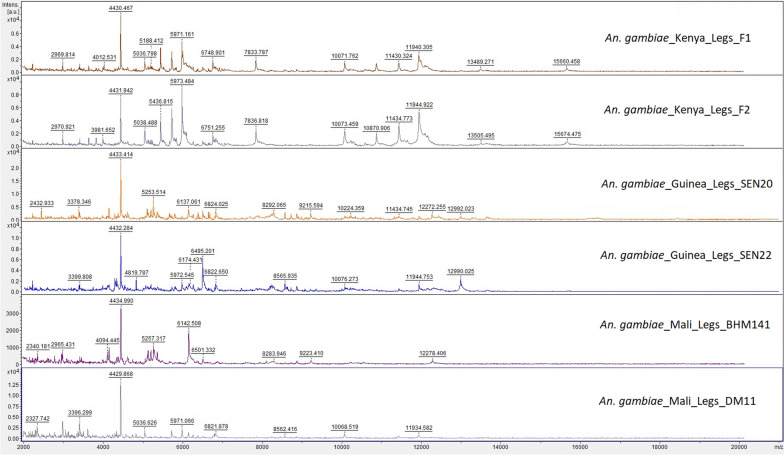
Representative mass spectrum protein profiles of legs of *Anopheles gambiae* from Kenya, Mali and Guinea. Specimens from Kenya are laboratory-reared females (mass spectra library). Specimens from Mali and Guinea are field-caught females (panel A and panel B, respectively). *A.u* arbitrary units; *m*/*z* mass to charge ratio in Daltons, *F* female

**Fig. 8:**
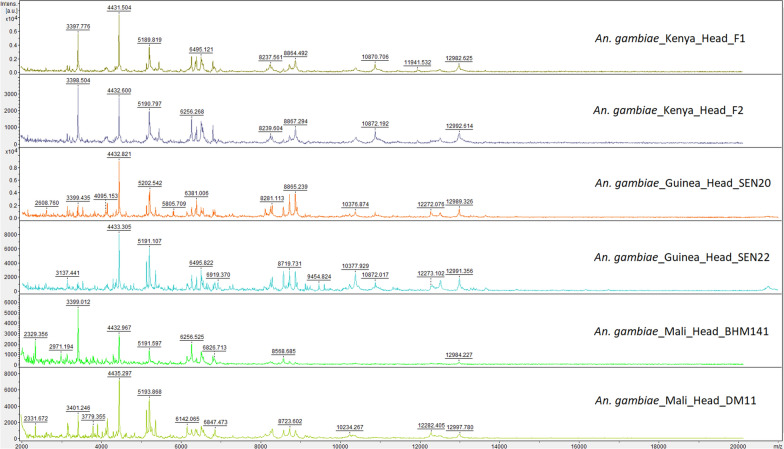
Representative mass spectrum protein profiles of head of *Anopheles gambiae* from Kenya, Mali and Guinea. Specimens from Kenya are laboratory-reared females (mass spectra library). Specimens from Mali and Guinea are field-caught females (panel A and panel B, respectively). *A.u* arbitrary units, *m*/*z*, mass to charge ratio in Daltons, *F* female

The mass spectrum protein profiles from legs and head of *An. gambiae* from Kenya (MSL), Mali and Guinea (panel A and panel B, respectively) are shown in Figs. 7 and 8. One identical peak was observed between the three geographical origins (*m*/*z* 4430) using the two body parts. The legs spectra from Kenya showed numerous differential peaks with that from the other sites, which can explain the low rate of correct identifications using database 1. With the head, spectra were more homogeneous across the different sites and few differential peaks were visible. The head showed a higher number of shared peaks between the different origins (*m*/*z* 3399, *m*/*z* 5190, *m*/*z* 8864 for instance) which can explain the better performances compared to the legs. Using a dendrogram (Additional files 9, 10: Figs. S9, S10), some clusters of spectra of the same geographic origin have been observed. However, other spectra from different origin were also grouped in similar branches, despite the high geographical distance between them.

## Discussion

This study provides new insight into the use of MALDI-TOF MS for *Anopheles* species identification. It tested the best-suited body part and the impact of the geographic origin of the specimens using two independent panels from different mosquito populations and four databases.

Differences of mass spectrum protein profiles and reproducibility levels were observed between body parts of *Anopheles* species from MSL and panels. The spectra from the legs exhibited the smaller number of peaks of high intensity, showing that the protein content was less diverse than for the head and thorax. Previous studies concluded that legs provided sufficient protein material to give reproducible and specific mass spectra [10–14]. However, a recent study reported that the using of less than four legs could compromise the MALDI-TOF MS identification of mosquito species, showing that at least four legs are required to get sufficient protein material [25]. In addition, one of the previous studies observed that the quality of legs spectra from field-caught *Anopheles* was lower than that from colony specimens, with a decreased intensity [14]. This suggests a possible protein degradation of the legs from field-caught specimens. As previously mentioned [16], the fragility of the legs, which are breakable and can be lost during collection, transportation, storage or processing, may lead to partial or total loss of the protein content. Indeed, a study showed that legs were prone to degradation during the trapping, with modification of protein profiles and a decrease of identification log(scores) as the trapping duration increased, even after 24 h of trapping [25]. Similarly, disparities have been observed in this study, between spectra from laboratory-reared specimens and from field-caught specimens for every anatomic parts. Field-caught specimens showed lower reproducibility levels. In addition, the duration of storage also seems to have impacted the mass spectra reproducibility levels. Indeed, the spectra of colony specimens of *An. arabiensis* obtained after the shortest storage duration (3 weeks at − 20 °C) had high reproducibility levels for every anatomic parts, contrary to the other colony specimens of the MSL. The head provided the highest reproducibility of mass spectra, no matter the origin of the specimens (colony or field) and no matter the conditions of storage, compared to the legs and thorax. This was consistent with the presence of higher identification log(scores) using the head. Therefore, the head protein content could be less prone to degradation and more robust than the other body parts.

This study has revealed that *Anopheles* thorax spectra from engorged field-caught specimens dissected after frozen storage were negatively impacted by the blood meal, contrary to the head and the legs ones. Two previous studies [15, 16] used the cephalothorax, as it gave a stronger mass spectrometry signal than the legs and provided the minimum concentration of 0.2 mg/mL raw protein recommended by Steinmann et al. [26]. However, the majority of specimens included were laboratory-raised from larvae field-collected and were non-engorged. One of the two studies also included resting females caught by aspiration and potentially blood-fed [15]. The number of peaks from specimens caught by aspiration was lower than that from specimen’s laboratory-raised and sometimes no peaks were observed. Thus, they postulated that the abdomen blood content somehow negatively influenced the frozen preservation of the engorged specimens. Similarly, for MALDI-TOF identification of sand fly species, the thorax of engorged specimens led to blood contaminations during the separation from the abdomen, after frozen storage [27–29]. Here, visually engorged *Anopheles* displayed specific patterns in thorax protein profiles and mass spectra reproducibility level of field-caught specimens was lower to that of laboratory-reared ones. These protein patterns probably correspond to haemoglobin signal, modified after blood digestion process and /or frozen storage. To precisely identify the proteins, this would require the using of other proteomic tools such as LC/MS. In contrast, Vega Rua et al*.* [30] observed highly reproducible thorax spectra of *Aedes* sp. and *Culex* sp, both laboratory-reared and field-caught, using frozen storage at − 20 °C from a few months to one year. However, the authors included only non-engorged female mosquitoes. Other field parameters can impact the *Anopheles* protein content and led to heterogeneity of mass spectra between laboratory-reared and field-caught specimens. For instance, seasonal fluctuations in temperature can also modify the phenotype. In a field population of *Anopheles merus* captured in South Africa, the mean wing length decreased by 19.6% in summer [31]. This illustrates the benefit of adding a high diversity of field-caught *Anopheles* in validation panels and spectral databases.

Using the initial MSL that did not contain specimens of the same origin and storage conditions as the panels, the proportion of interpretable (LSV ≥ 1.7) and correct identifications was significantly higher using the head than using the thorax or the legs. However, this proportion of correct identifications remained low (64%) during the query of panels (A+B). Using the head, the proportion of specimens with an LSV < 1.7 was of 16% whereas greater proportions were observed for the thorax (58%) and the legs (33%). In contrast, using database 2, containing specimens of the same origin and storage conditions as the panel A, the results were significantly improved. Indeed, the legs provided high proportions of correct identifications, comparable to the head (96% and 98%, respectively), which was in agreement with the previous studies using the legs that also included specimens of the same origin as the panels into the databases [10–14]. Using database 4 that contained additional specimens from Senegal, the difference between the proportions of correct identifications using legs and head was higher (96% and 92%, respectively) but remained not significant. Nevertheless, by comparing the results according to the number of deposits of protein extracts, significant differences between legs and head have been shown. The head provided better performances compared to the legs as it did not require the deposit of multiple spots to optimize the log(score) results. Indeed, with consideration of the highest scoring spectrum, the legs required the using of four replicates of protein extract whereas for the head, only one replicate provided almost equivalent results as using four replicates, at the LSV threshold of 1.7 and 1.8. These results are concordant with a recent study on mosquitoes [25] that observed that using the legs, the LSVs were improved when three spots of each sample were deposited onto the target plate, compared to the using of only one spot. Therefore, the using of the head will represent a concrete improvement of the routine use of MALDI-TOF for *Anopheles* identification, as it will allow to gain rapidity of analysis by decreasing the number of deposits. For the thorax, the proportion of correct identifications significantly increased (81%) using panel A against database 2, but remained lower than for the other body parts. A database associating thorax, head and legs spectra may improve the identification results, especially when only legs are used for database queries. Indeed, 46.7% of the leg spectra of panel A had cross-matching with the head or thorax spectra of the database 1. The same observation was made using panel A against database 2 (41.3% of cross-matching using the legs). This result was consistent with Vega Rua et al*.* [30], who recommended a double database creation with thorax and legs to improve the identification of specimens with missing or damaged legs. For database querying, they also recommended the use of both the thorax and legs for double checking of mosquito species identification. Here, for database querying using a database without specimens of the same origin as the tested ones, superiority was observed using the head alone instead of using “thorax + legs” or other associations of body parts.

The potential of including *Anopheles* specimens from the geographic area to be investigated has been confirmed. This reflects a great heterogeneity of mass spectra protein profiles between the *Anopheles* specimens of the initial MSL and specimens of the panels. As all the previous studies have included in the databases specimens of the same origin as the specimens to be tested, they did not reveal as much the importance of this methodology. However, heterogeneity of mass spectra was also reported when comparing mosquito species [10, 11, 16] or sand fly species [22, 32, 33] from various geographical origins and between reared and field mosquito spectra [10, 15]. For a same species, the observation of biomarkers specific to colony specimens and to field specimens [10, 15] suggests a great variability in protein content due to phenotypic distinctness in relation to the genetic diversity of *Anopheles,* influenced by environmental settings, evolutionary history adaptation, demographic history or genetic drift. However, clustering analyses indicates that the experimental conditions seem to also have a great impact on mass spectrum protein profiles. In this study, the mass spectra protein profiles of *An. gambiae* from Kenya, Mali and Guinea have been compared and the spectra were not exclusively clustered according to the geographical origin in a dendrogram. Similarly, a study reported that specimens both from the same *Anopheles* species and colony were split in different groups of a dendrogram [15]. Therefore, it is supposed that variability of mass spectra can also result from the method of storage or other experimental conditions such as trapping method or trapping duration [25], quality of protein extraction and homogenization [34]. In addition, even if we did not know precisely the age of the colonies, as there was no clear clustering of the spectra from colony specimens, it probably not has impacted the results. These variations between findings may pose a challenge in practical use of MALDI TOF MS for mosquitoes’ identification and may complicate the creation of large international databases, in contrast to bacteria or fungi. Region-specific mass spectrum databases will have to be produced. Moreover, important efforts of standardization will be necessary, such as the using of internal biomarkers, as previously suggested [10, 15].

Most identification errors consisted of mismatches between the cryptic species *An. gambiae* and *An. arabiensis,* which are well described in the Gambiae complex [10, 11, 15]. The identification of the cryptic species seems to be even more susceptible to the experimental conditions and database species composition. Indeed, the addition in the database of specimens of the same origin as that of the panel significantly decreased the mismatch between *An. arabiensis* and *An. gambiae*. It is not surprising, as a previous study observed only four identical biomarkers between laboratory-reared and field-caught *An. arabiensis* specimens [10]. However, the addition of close species into the databases, such as specimens of *An. arabiensis* field-caught from Senegal in the Databases 3 and 4, increased identification errors using the head and the legs, but not significantly. Between field-caught *An. arabiensis* and *An. gambiae,* identification mismatches have been reported using the legs, even with an LSV > 2 [10]. The authors have reported 19 identical peaks masses between field-caught *An. gambiae* and *An. arabiensis* for the spectra of legs, explaining the mismatches. They pointed out the limitations of usual bio-informatic tools in distinguishing clearly between cryptic species. Similarly, another study has shown that the cryptic species of the Gambiae complex, including *An. arabiensis* and *An. gambiae* did not segregate into well-defined clusters in a dendrogram [15]. Using the cephalothorax, the presence of biomarkers specific to each species of the Gambiae complex allowed classification of mass spectra using machine learning methods, opening the door to new approaches.

A limitation of the study is that some results may have been affected by the use of various storage methods and the duration of storage. Indeed, some specimens were dry frozen preserved and analysed several months or years later, whereas other specimens were stored at ambient temperature and analysed in a few weeks. However, as these various storage conditions have been shown to preserve the quality of spectra, the results were most likely only partially affected [8, 25]. Another limitation is that only one field-caught species was tested in the panels, which was the dominant species *An. gambiae*. Further studies using larger databases and panels exhibiting more species diversity are required, especially to improve the resolution of MALDI-TOF MS for closely related species. MALDI-TOF MS should be a good alternative to molecular methods for eco-epidemiological studies of *Anopheles* vectors when taxonomic resolution is adequate. The technique does not require much training, in contrast to the morphological identification of *Anopheles*. In addition, MALDI-TOF MS analyses of one hundred specimens can be assessed in a few hours, whereas molecular methods require several steps of analysis, from DNA extraction to sequence editing and assignment. Once the MALDI-TOF MS instrument is acquired, which is expensive and therefore a major investment ($200,000 for a complete system), this method requires inexpensive consumables, and the cost is estimated at $1–2 per sample. It may be useful in areas where entomological experts may not be available, for damaged specimens and to distinguish cryptic species. Similar to DNA sequence databases, large use of MALDI-TOF MS databases requires accessibility through online applications, as previously remarked [8, 9, 22, 32]. Such online platforms have already been proposed for fungi [23] and *Leishmania* species [35]. Therefore, we plan to share an MSL dedicated to *Anopheles* species identification via an online platform that is currently being set up, following a suggestion by Schaffner et al*.* for mosquito surveillance [36].

## Conclusions

The protein repertoire of *Anopheles* varied according to compartment, which impacted the performances of species identification using MALDI-TOF MS. The head provided the most robust protein content compared to the legs and the thorax. The head spectra showed the best performances, allowing the using of less than four replicates of protein extract. The thorax of engorged specimens may not be used alone due to the possibility of interactions with the abdomen content after frozen storage. Variations between findings may complicate the creation of large international databases and region-specific mass spectrum databases will have to be produced. This study is a new step towards an optimization of MALDI-TOF MS for *Anopheles* species identification. However, further research is needed to improve the resolution for cryptic species using new bio-informatic tools and for a better standardization in order to share online spectral databases.

## Supplementary Information

**Additional file 1**: **Fig. S1**. Maximum likelihood tree of *Anopheles* ITS2 sequences. Seaview v4 software, Clustal W and phyML tools. Specimens identified in reference centres are indicated by taxonomic identification along with GenBank accession number, namely, *An. arabiensis* (Pretoria University, South Africa), *An. gambiae* (IRD Montpellier, France) and *An. funestus* (MRTC Bamako, Mali).

**Additional file 2**: **Fig. S2**. Impact of the log(score) threshold on MALDI-TOF MS species identification using panel A from Mali versus database 1 for each body part of *Anopheles*, n=52. The number of specimens having correct species identification, error of species identification and absence of identification due to an LSV<threshold are shown in different colours for each body part.

**Additional file 3**: **Fig. S3**. Composite correlation index (CCI) heat map grid of mass spectrum protein profiles of *Anopheles gambiae.* Panel A from Mali, n=52 (a). Panel B from Guinea, n=40 (b). Levels of mass spectral reproducibility are indicated in blue and red, revealing incongruence and relatedness between spectra, with a correlation index variation between 0 and 1, respectively. The coloured squares of the central diagonal reflect the degree of reproducibility of each mass spectrum when compared to itself. Around the central diagonal, spectra from various specimens of *Anopheles gambiae* were compared. The CCI matrix was calculated using MALDI Biotyper v4.1 software with default settings.

**Additional file 4**: **Fig. S4**. Impact of body part on identification results using panels A+B versus database 1, n=92. The number of specimens having correct species identification, error of species identification and absence of identification due to an LSV<1.7 are shown in different colours for each body part.

**Additional file 5**: **Fig. S5**. Cross-matching between anatomic parts and sex, panel A versus database 1, n=52. The number of specimens of panel A is shown on the vertical axis. Characteristics of the corresponding MSPs of database 1 (anatomic parts, sex and insufficient matching due to LSV <1.7) are shown in different colours.

**Additional file 6**: **Fig. S6**. Impact of the association of anatomic parts, panel A versus database 1, n=52. The number of specimens having correct species identification, error of species identification and absence of identification due to an LSV<1.7 are shown in different colours for each body part and association of body parts.

**Additional file 7**: **Fig. S7**. Distribution of spectral log(scores) from heads, thoraces and legs. Panel A versus database 1 or database 2, n=52. Violin plots showing the distribution taking into account the densities of the points for the different log(score) values. The median score is represented with dashes, and the quartiles are represented by dashed lines.

**Additional file 8**: **Fig. S8**. Identification results, panel A versus database 1, database 2, database 3 or database 4, n=52. Database 1 was created using n=20 non-engorged laboratory-reared *Anopheles* and field specimens from the collection of reference centres. Database 2 was created by adding 10 *Anopheles* specimens collected from the field in Mali to database 1. Databases 3 and 4 were created by adding 10 field specimens from Senegal to databases 1 and 2, respectively. The number of specimens having correct species identification, error of species identification and absence of identification due to an LSV<1.7 are shown in different colours for each body part.

**Additional file 9**: **Fig. S9**. Dendrogram of legs mass spectra constructed with specimens of *Anopheles gambiae* from Kenya, Mali and Guinea (n=15). Specimens from Kenya are laboratory-reared females (mass spectra library). Specimens from Mali and Guinea are field-caught females (panel A and panel B, respectively). The dendrogram was calculated using MALDI Biotyper v4.1 and distance units correspond to relative similarity of mass spectra.

**Additional file 10**: **Fig. S10**. Dendrogram of head mass spectra constructed with specimens of *Anopheles gambiae* from Kenya, Mali and Guinea (n=15). Specimens from Kenya are laboratory-reared females (mass spectra library). Specimens from Mali and Guinea are field-caught females (panel A and panel B, respectively). The dendrogram was calculated using MALDI Biotyper v4.1 and distance units correspond to relative similarity of mass spectra.

## Data Availability

The datasets used and/or analysed during the current study are available from the corresponding author on reasonable request.

## Abbreviations

MALDI-TOF: Matrix-assisted laser desorption and ionization time of flight
MS: Mass spectrometry
MSL: Mass spectral library
MSP: Main spectrum profile
CCI: Composite correlation index
LSV: Log(score) value
ITS2: Internal transcribed spacer region 2
