# Supplementary figures and images for: New assessment of Anopheles vector species identification using MALDI-TOF MS
Source: Malar J. 2021 Jan 9;20:33. doi: 10.1186/s12936-020-03557-2 (PMC7796594; doi:10.1186/s12936-020-03557-2)

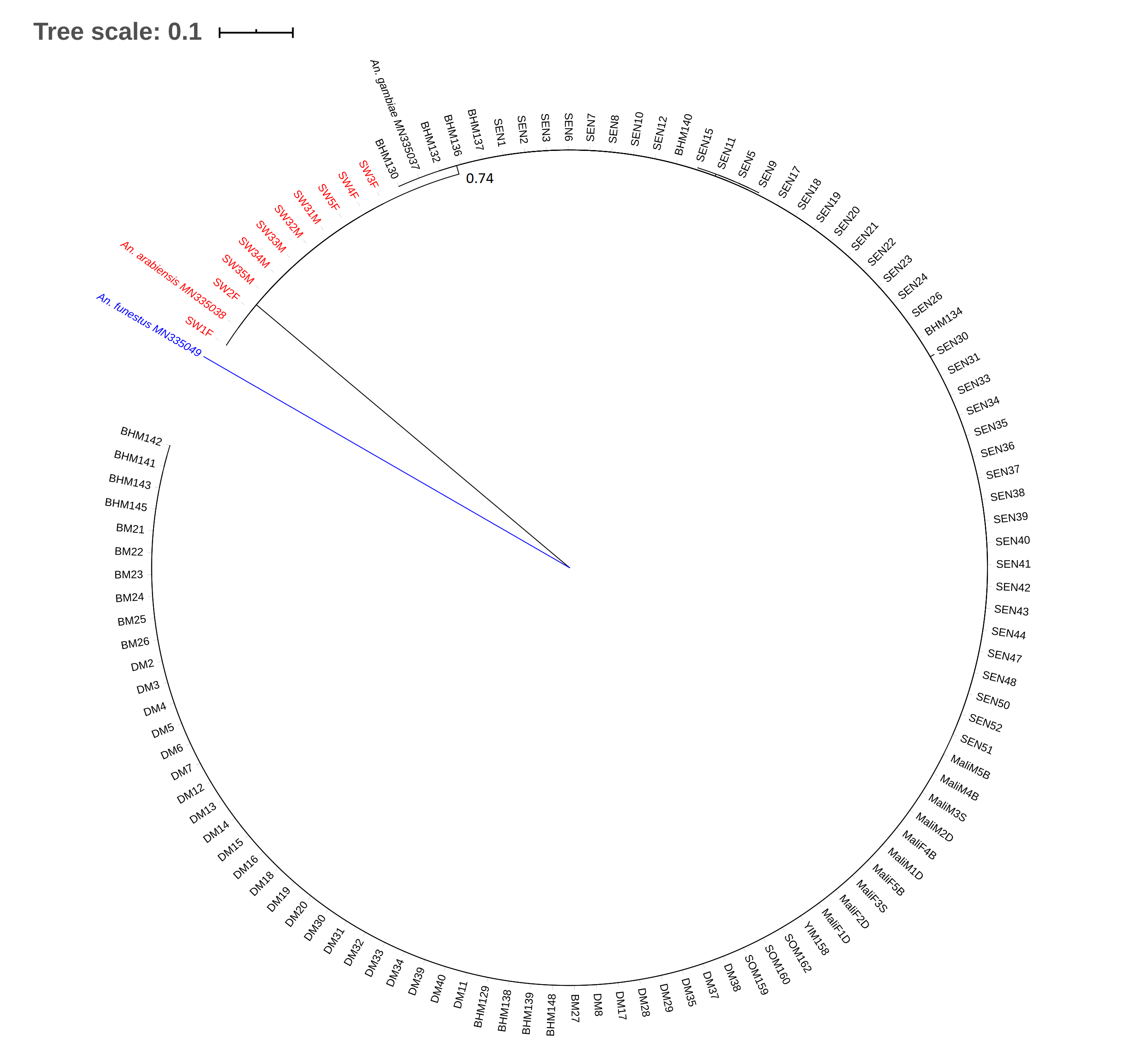

Supplement: Supplementary file 1 — Additional file 1: Fig. S1. Maximum likelihood tree of Anopheles ITS2 sequences. Seaview v4 software, Clustal W and phyML tools. Specimens identified in reference centres are indicated by taxonomic identification along with GenBank accession number, namely, An. arabiensis (Pretoria University, South Africa), An. gambiae (IRD Montpellier, France) and An. funestus (MRTC Bamako, Mali). [file 12936_2020_3557_MOESM1_ESM.png]

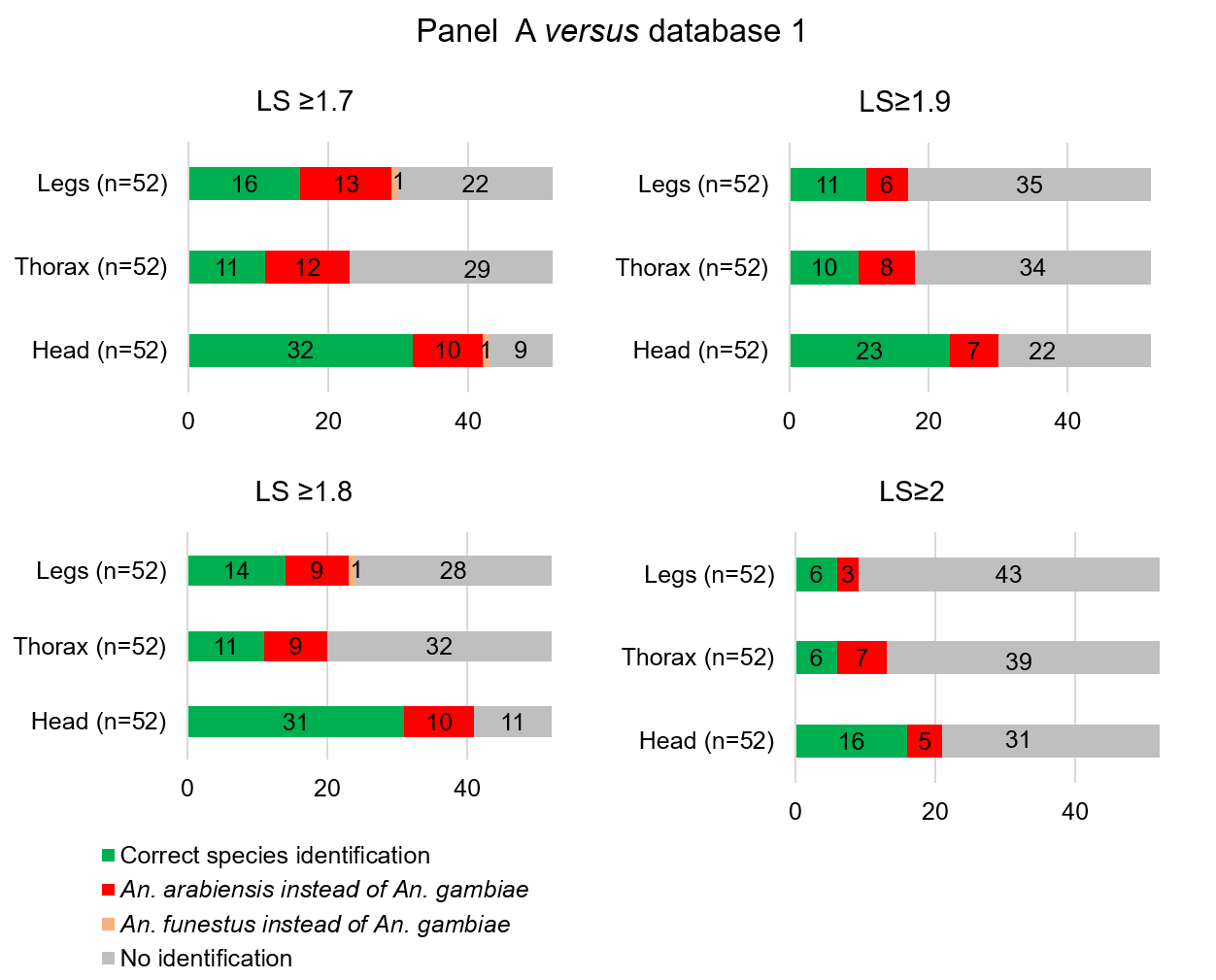

Supplement: Supplementary file 2 — Additional file 2: Fig. S2. Impact of the log(score) threshold on MALDI-TOF MS species identification using panel A from Mali versus database 1 for each body part of Anopheles, n=52. The number of specimens having correct species identification, error of species identification and absence of identification due to an LSV<threshold are shown in different colours for each body part. [file 12936_2020_3557_MOESM2_ESM.png]

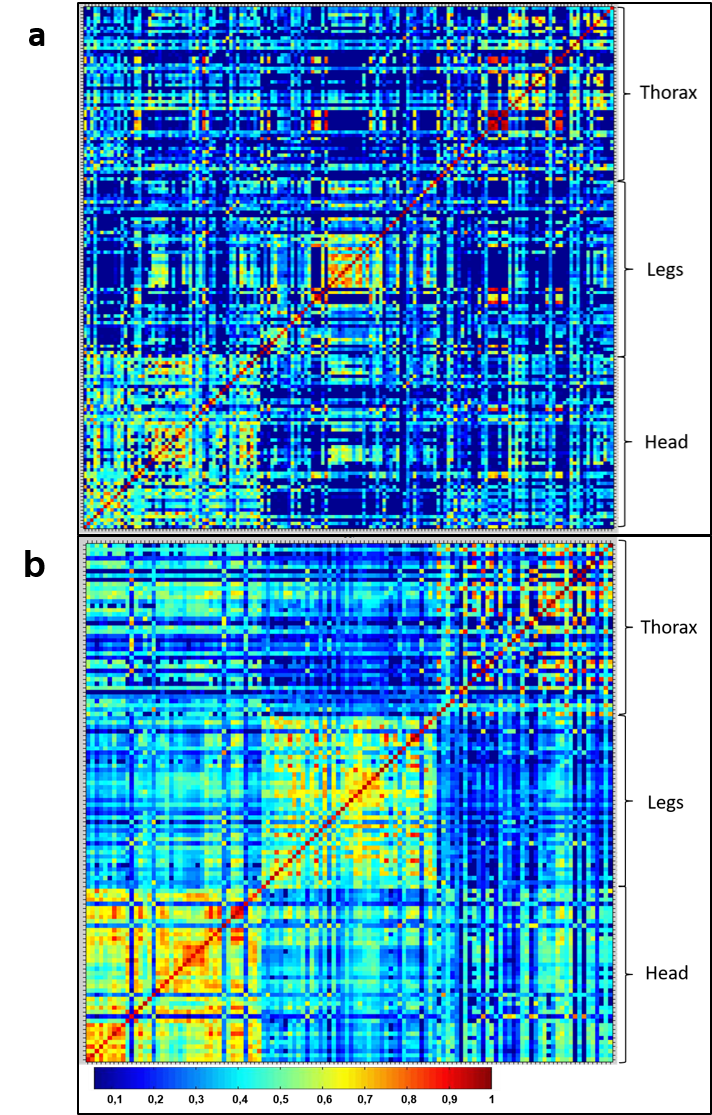

Supplement: Supplementary file 3 — Additional file 3: Fig. S3. Composite correlation index (CCI) heat map grid of mass spectrum protein profiles of Anopheles gambiae. Panel A from Mali, n=52 (a). Panel B from Guinea, n=40 (b). Levels of mass spectral reproducibility are indicated in blue and red, revealing incongruence and relatedness between spectra, with a correlation index variation between 0 and 1, respectively. The coloured squares of the central diagonal reflect the degree of reproducibility of each mass spectrum when compared to itself. Around the central diagonal, spectra from various specimens of Anopheles gambiae were compared. The CCI matrix was calculated using MALDI Biotyper v4.1 software with default settings. [file 12936_2020_3557_MOESM3_ESM.png]

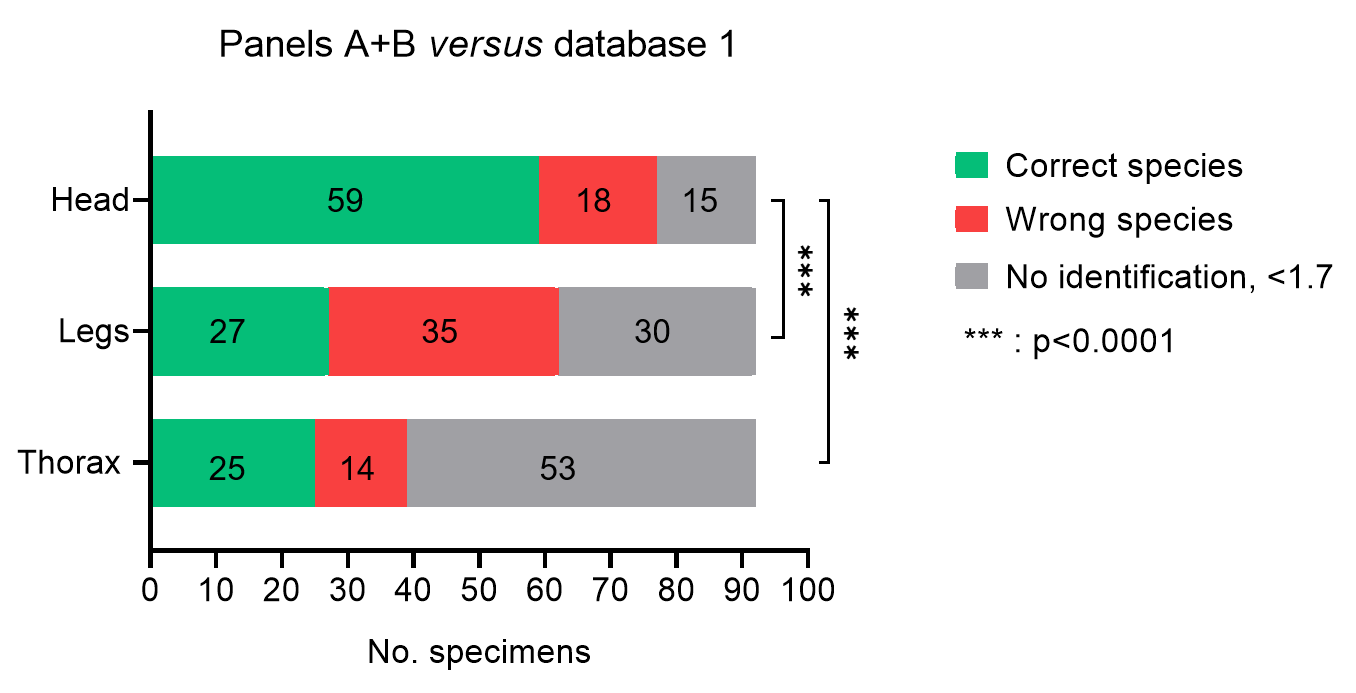

Supplement: Supplementary file 4 — Additional file 4: Fig. S4. Impact of body part on identification results using panels A+B versus database 1, n=92. The number of specimens having correct species identification, error of species identification and absence of identification due to an LSV<1.7 are shown in different colours for each body part. [file 12936_2020_3557_MOESM4_ESM.png]

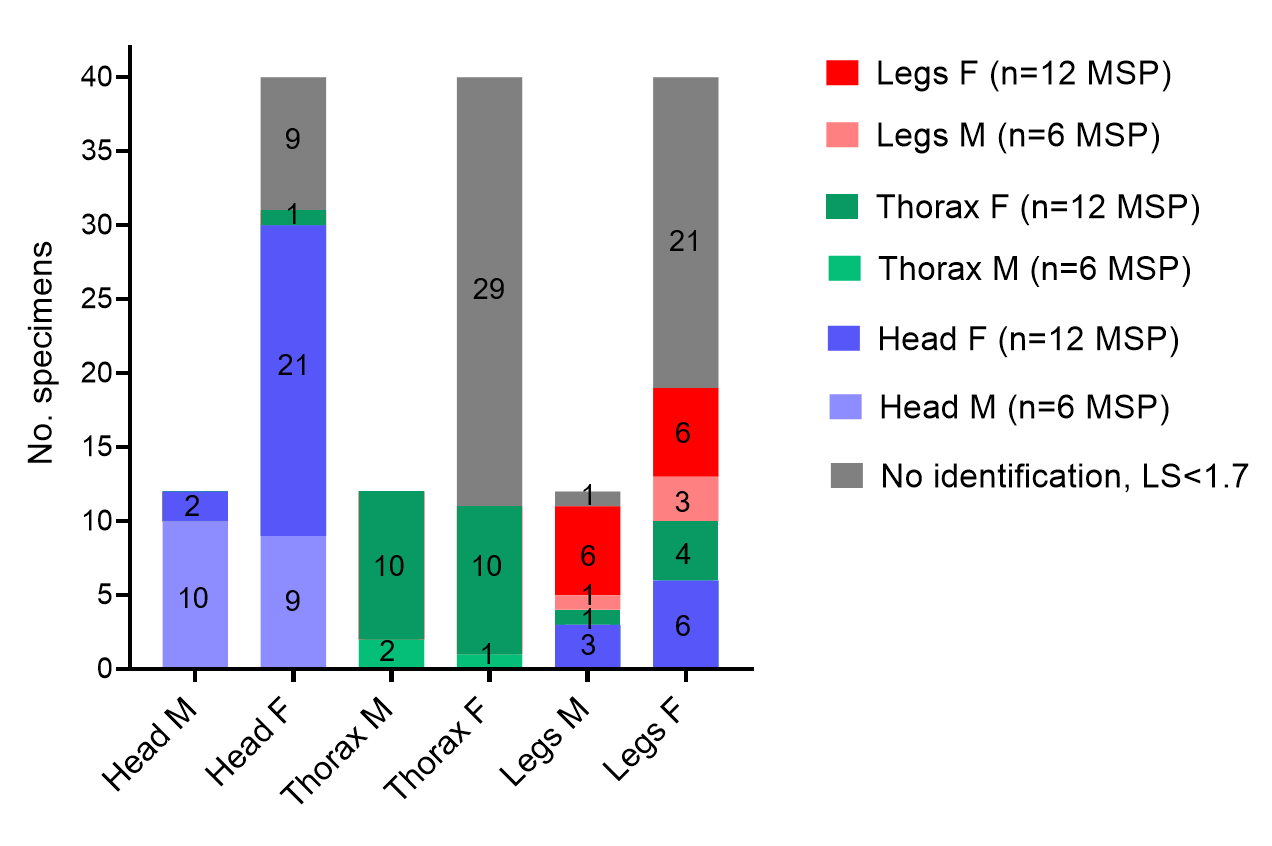

Supplement: Supplementary file 5 — Additional file 5: Fig. S5. Cross-matching between anatomic parts and sex, panel A versus database 1, n=52. The number of specimens of panel A is shown on the vertical axis. Characteristics of the corresponding MSPs of database 1 (anatomic parts, sex and insufficient matching due to LSV <1.7) are shown in different colours. [file 12936_2020_3557_MOESM5_ESM.png]

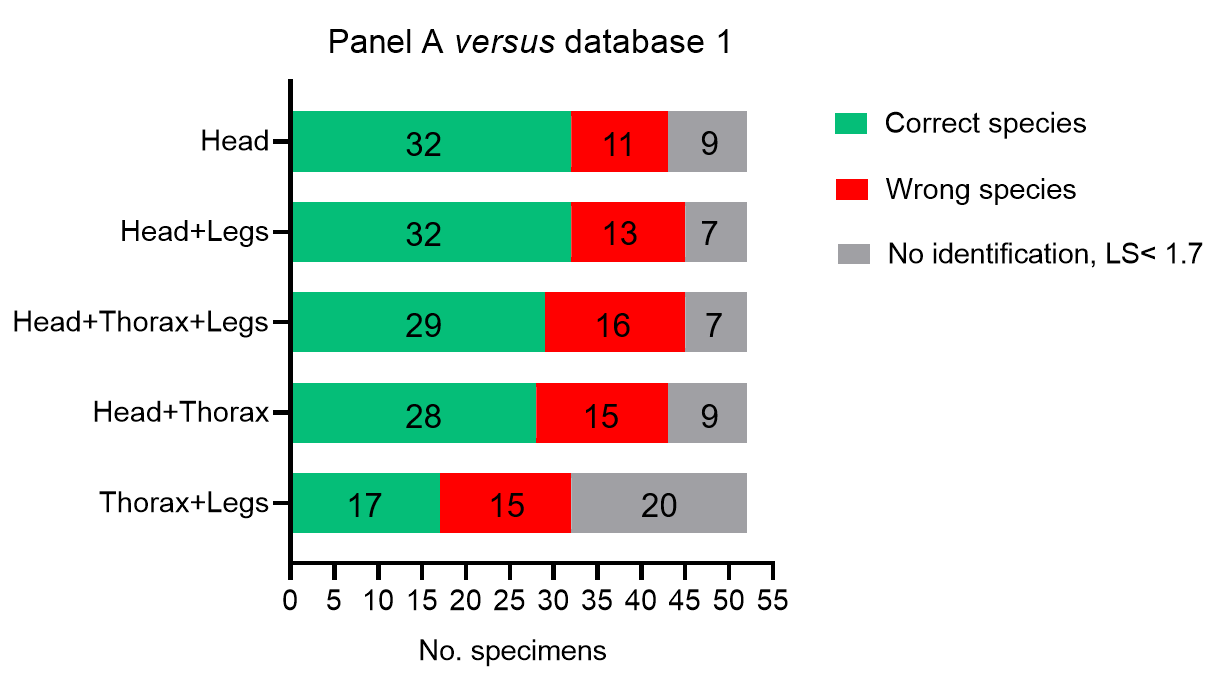

Supplement: Supplementary file 6 — Additional file 6: Fig. S6. Impact of the association of anatomic parts, panel A versus database 1, n=52. The number of specimens having correct species identification, error of species identification and absence of identification due to an LSV<1.7 are shown in different colours for each body part and association of body parts. [file 12936_2020_3557_MOESM6_ESM.png]

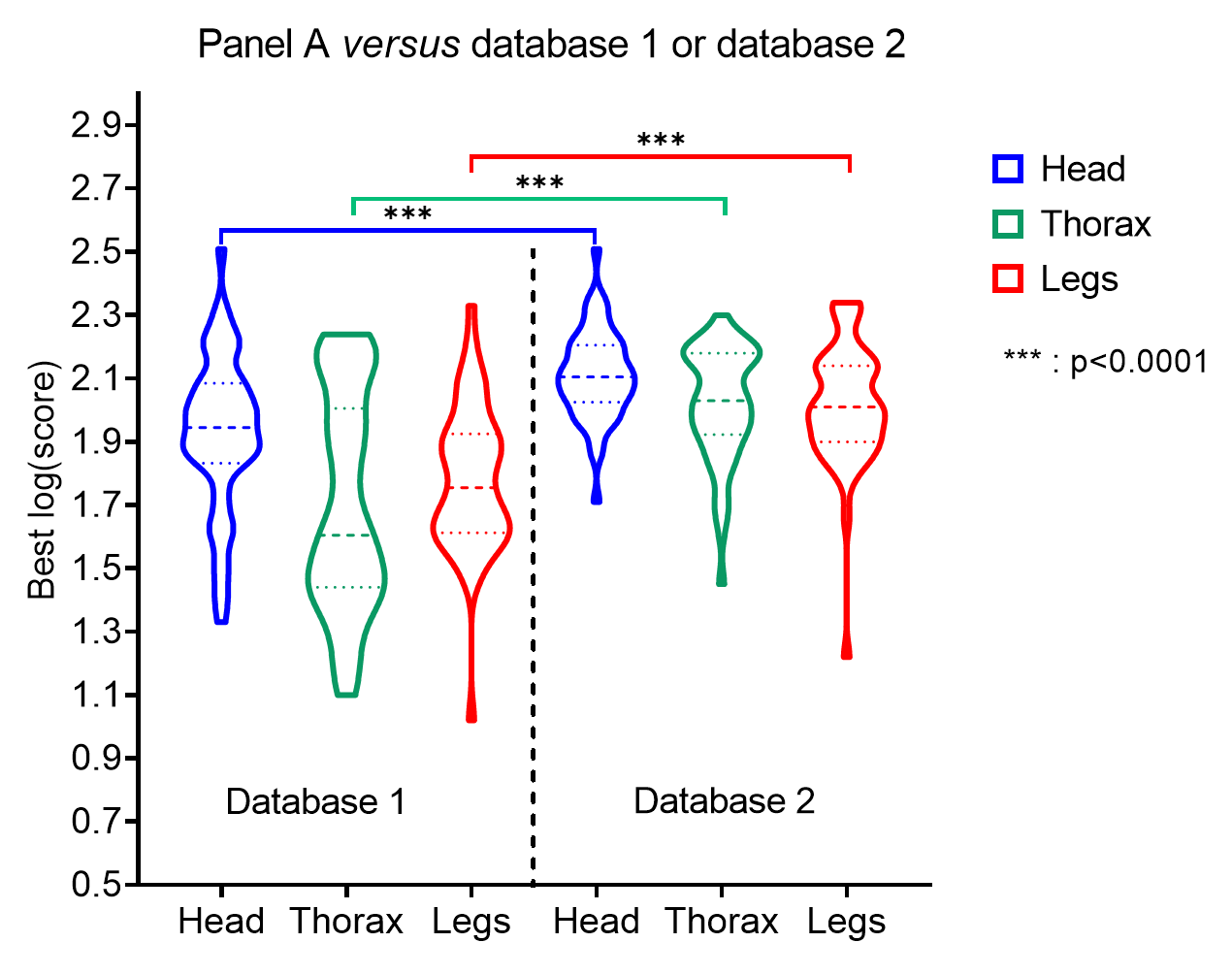

Supplement: Supplementary file 7 — Additional file 7: Fig. S7. Distribution of spectral log(scores) from heads, thoraces and legs. Panel A versus database 1 or database 2, n=52. Violin plots showing the distribution taking into account the densities of the points for the different log(score) values. The median score is represented with dashes, and the quartiles are represented by dashed lines. [file 12936_2020_3557_MOESM7_ESM.png]

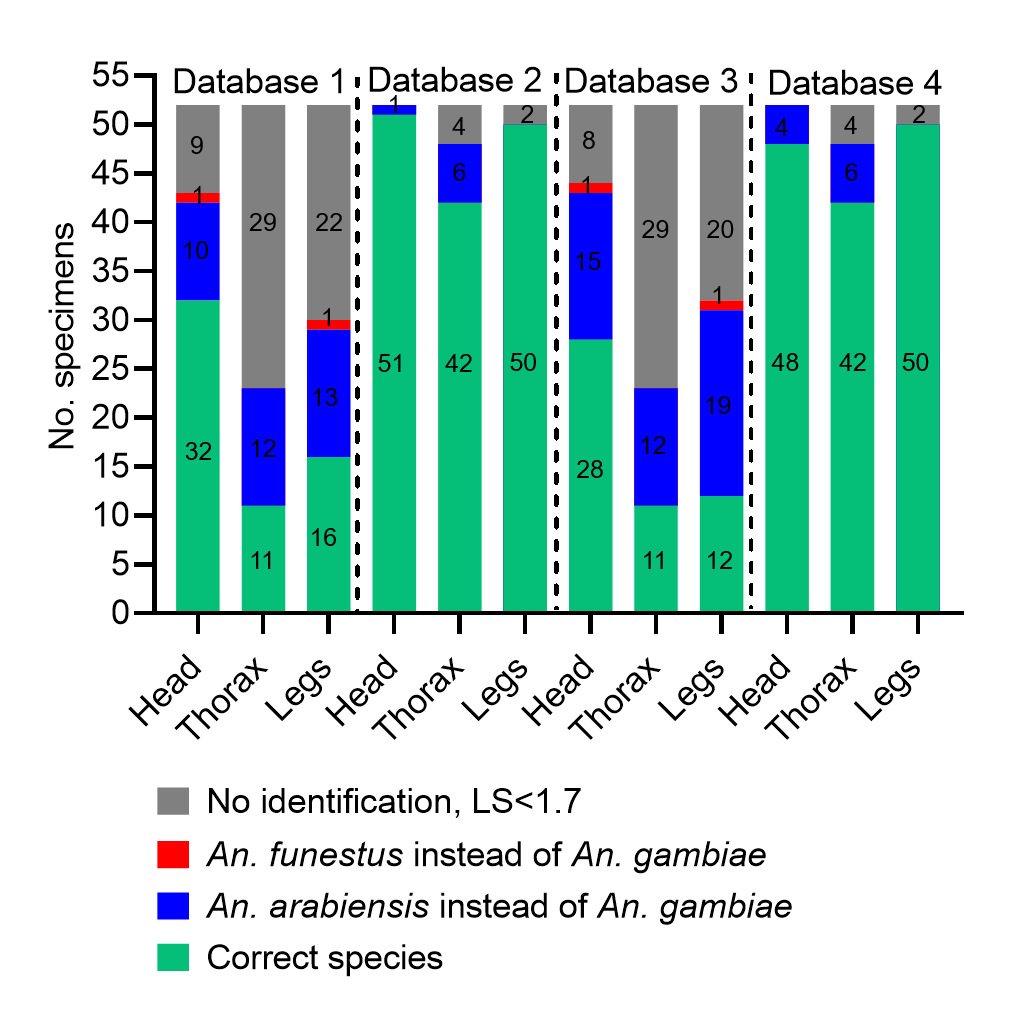

Supplement: Supplementary file 8 — Additional file 8: Fig. S8. Identification results, panel A versus database 1, database 2, database 3 or database 4, n=52. Database 1 was created using n=20 non-engorged laboratory-reared Anopheles and field specimens from the collection of reference centres. Database 2 was created by adding 10 Anopheles specimens collected from the field in Mali to database 1. Databases 3 and 4 were created by adding 10 field specimens from Senegal to databases 1 and 2, respectively. The number of specimens having correct species identification, error of species identification and absence of identification due to an LSV<1.7 are shown in different colours for each body part. [file 12936_2020_3557_MOESM8_ESM.png]

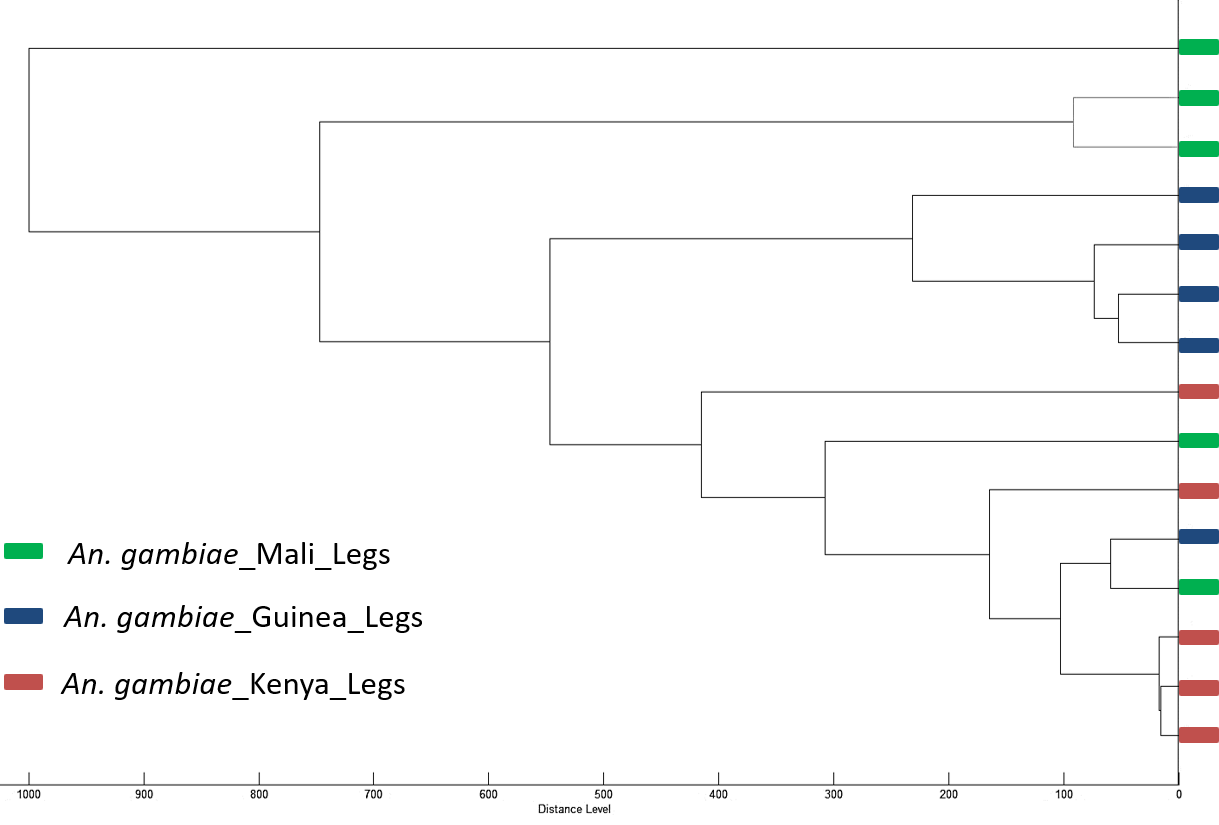

Supplement: Supplementary file 9 — Additional file 9: Fig. S9. Dendrogram of legs mass spectra constructed with specimens of Anopheles gambiae from Kenya, Mali and Guinea (n=15). Specimens from Kenya are laboratory-reared females (mass spectra library). Specimens from Mali and Guinea are field-caught females (panel A and panel B, respectively). The dendrogram was calculated using MALDI Biotyper v4.1 and distance units correspond to relative similarity of mass spectra. [file 12936_2020_3557_MOESM9_ESM.png]

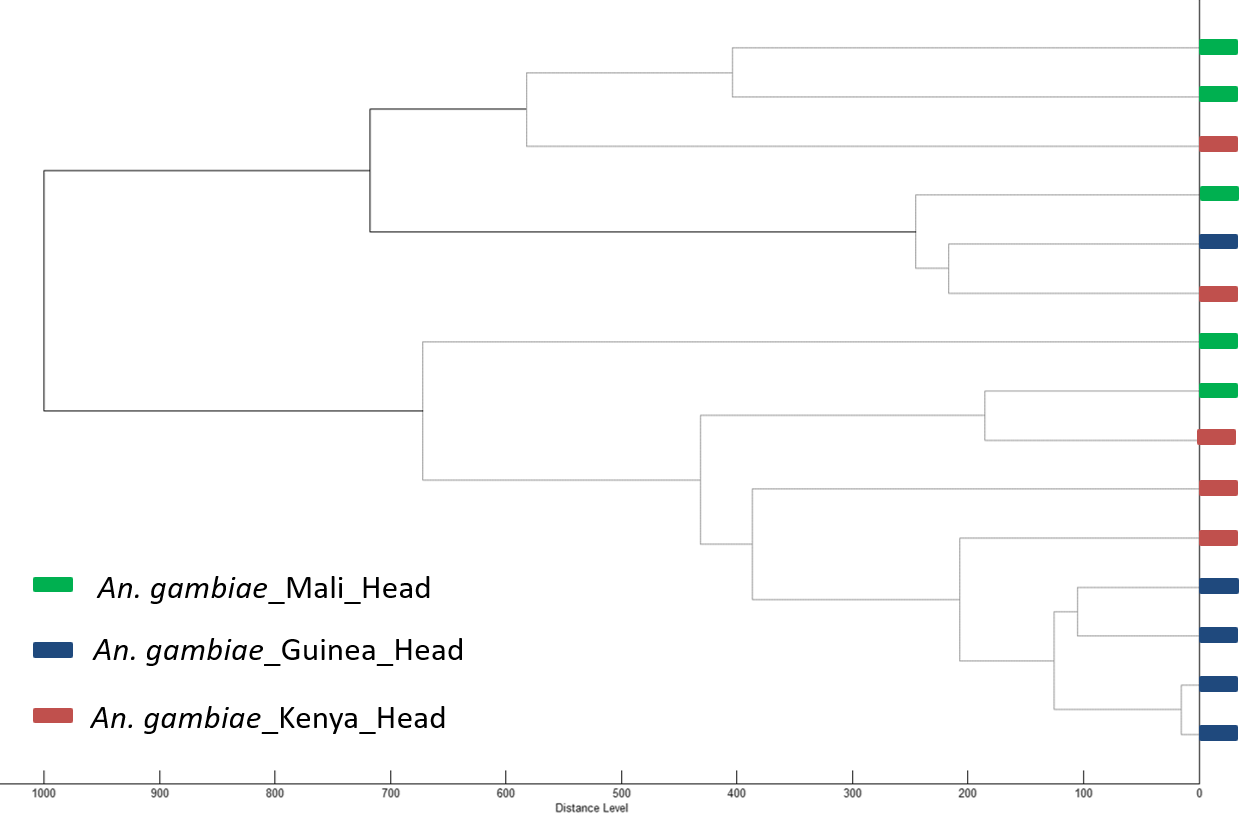

Supplement: Supplementary file 10 — Additional file 10: Fig. S10. Dendrogram of head mass spectra constructed with specimens of Anopheles gambiae from Kenya, Mali and Guinea (n=15). Specimens from Kenya are laboratory-reared females (mass spectra library). Specimens from Mali and Guinea are field-caught females (panel A and panel B, respectively). The dendrogram was calculated using MALDI Biotyper v4.1 and distance units correspond to relative similarity of mass spectra. [file 12936_2020_3557_MOESM10_ESM.png]
